# Lactate metabolism in human health and disease

**DOI:** 10.1038/s41392-022-01151-3

**Published:** 2022-09-01

**Authors:** Xiaolu Li, Yanyan Yang, Bei Zhang, Xiaotong Lin, Xiuxiu Fu, Yi An, Yulin Zou, Jian-Xun Wang, Zhibin Wang, Tao Yu

**Affiliations:** 1 Center for Regenerative Medicine, Institute for Translational Medicine, The Affiliated Hospital of Qingdao University; Department of Cardiac Ultrasound, The Affiliated Hospital of Qingdao University, No. 16 Jiangsu Road, Qingdao, 266000 China; 2 Department of Immunology, School of Basic Medicine, Qingdao University, Qingdao, 266071 China; 3 Department of Respiratory Medicine, Qingdao Municipal Hospital, Qingdao, 266011 China; 4 Department of Cardiac Ultrasound, The Affiliated Hospital of Qingdao University, No. 16 Jiangsu Road, Qingdao, 266000 China; 5 Department of Cardiology, The Affiliated Hospital of Qingdao University, No. 1677 Wutaishan Road, Qingdao, 266555 China

**Keywords:** Epigenetics, Cancer

## Abstract

The current understanding of lactate extends from its origins as a byproduct of glycolysis to its role in tumor metabolism, as identified by studies on the Warburg effect. The lactate shuttle hypothesis suggests that lactate plays an important role as a bridging signaling molecule that coordinates signaling among different cells, organs and tissues. Lactylation is a posttranslational modification initially reported by Professor Yingming Zhao’s research group in 2019. Subsequent studies confirmed that lactylation is a vital component of lactate function and is involved in tumor proliferation, neural excitation, inflammation and other biological processes. An indispensable substance for various physiological cellular functions, lactate plays a regulatory role in different aspects of energy metabolism and signal transduction. Therefore, a comprehensive review and summary of lactate is presented to clarify the role of lactate in disease and to provide a reference and direction for future research. This review offers a systematic overview of lactate homeostasis and its roles in physiological and pathological processes, as well as a comprehensive overview of the effects of lactylation in various diseases, particularly inflammation and cancer.

## Introduction

Since its discovery in 1780, lactate has been often wrongly assumed to be a metabolic waste product under hypoxic conditions with multiple harmful effects and to be associated with low oxygen conditions^1^. The lactate shuttle hypothesis describes the roles of lactate in the delivery of oxidative and gluconeogenic substrates and cellular signaling^2^. Brooks’ research demonstrated how lactate is formed and utilized under completely aerobic conditions^3^. Evidence for lactate as a significant modulator of the coordination of systemic metabolism has grown immensely^4,5^. No longer considered a waste product of anaerobic metabolism, lactate is increasingly being explored as a signaling molecule. Lactate has been shown to signal through its specific receptor G protein-coupled receptor 81 (GPR81)^6^ or to be transported into cells by monocarboxylate transporters (MCTs)^7^.

In the 1920s, Otto Warburg observed for the first time that tumors consume more glucose than surrounding normal tissue, leading him to propose the phenomenon of aerobic glycolysis, wherein glucose can be fermented to produce lactate instead of carbon dioxide, even in the presence of oxygen; this phenomenon is now known as the Warburg effect. The most immediate consequences of aerobic glycolysis are increased intracellular and extracellular lactate concentrations^8^. Warburg’s original thesis delineates the process by which irreversible mitochondrial damage leads to aerobic glycolysis in tumor cells^9^. In contrast, cell metabolic reprogramming leading to the inhibition of mitochondrial oxidative phosphorylation (OXPHOS) is the primary factor underlying aerobic glycolysis in tumor cells^10,11^. The occurrence of aerobic glycolysis can be attributed to the increased metabolic demand for ATP in proliferative cells, such as tumor cells^12^. Therefore, glycolysis is highly vigorous in proliferative cells, ensuring higher intracellular and extracellular concentrations of lactate than those found in cells at the resting state. Notably, lactate accumulation in the tissue microenvironment is characteristic of inflammatory diseases and cancer^13^. Nevertheless, overwhelming evidence suggests the occurrence of the Warburg effect in many nontumor cells and in a wide range of noncancerous diseases, such as pulmonary hypertension, pulmonary fibrosis, heart failure, atherosclerosis, and polycystic kidney disease^14,15^. Compelling evidence indicates how aerobic glycolysis produces lactate under stressful conditions, such as trauma, infection, myocardial infarction, and heart failure. Lactate is always an inevitable end product of glycolysis, regardless of oxygen availability.

A study published in 2019 illustrated the important role of lactate in promoting the modification of histone lysine residues^16^. Similar to other posttranslational modifications (PTMs), such as acetylation, succinylation, and malonylation, lactylation leads to transcriptional regulation. Epigenetic modifications that drive metabolic regulation play significant roles in inflammation and cancer^17–20^. Hence, this review summarizes the biological functions of lactate and lactylation in regulating immune homeostasis and promoting tumor growth and emphasizes the importance of more comprehensive study of the other functions of lactate and lactylation. Therefore, in this unique review, the important roles of histone acetyltransferases (HATs) and histone deacetylases (HDACs) in the regulation of lactylation are elaborated. In summary, we present a comprehensive description of the important findings in the areas of lactate transport and signaling and other functions of lactate/lactylation in several pathophysiological processes and in specific diseases.

## Lactic acid homeostasis

### Lactic acid production and clearance

**Fig. 1:**
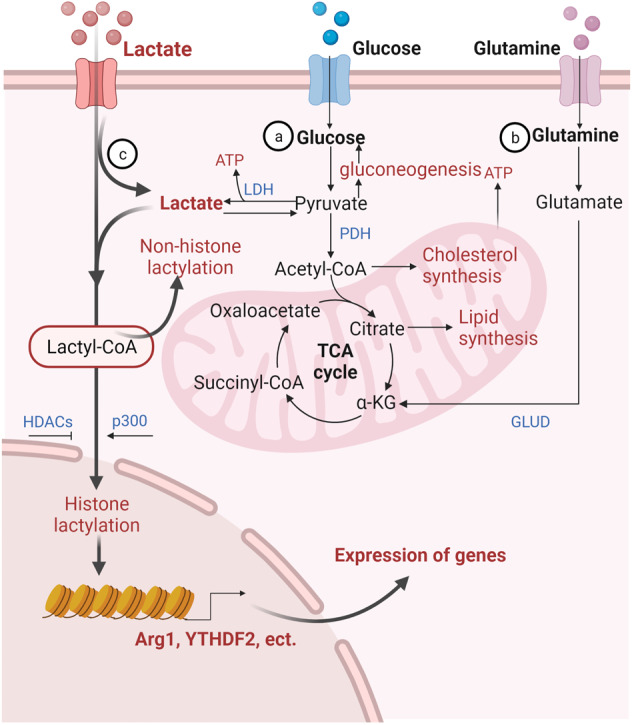
Lactate metabolism and lactylation in cells. In the cytoplasm, lactate is transported into cells by MCTs and is produced from glycolysis or glutamine decomposition. The catabolism of lactate in cells occurs through two pathways. In one pathway, lactate is oxidized to pyruvate, which enters mitochondria and is metabolized through the tricarboxylic acid cycle. In the other pathway, lactate is converted to glucose through gluconeogenesis. Lactate can be converted into lactyl-CoA and is involved in the lactylation of histones and nonhistone proteins. LDH lactate dehydrogenase; PDH pyruvate dehydrogenase; GLUD glutamate dehydrogenase; HDACs Histone Deacetylases. (Figure was created with Biorender.com.)

The production of lactate as fuel increases when the demand for oxygen and ATP exceeds the cellular supply, such as during strenuous exercise and infection^21,22^. Lactate is a classical byproduct of glucose metabolism, and the main lactate production pathway depends on glycolysis (Fig. 1). The glycolysis pathway is activated to compensate for a lack of ATP production when hypoxia inhibits the tricarboxylic acid (TCA) cycle. Specifically, glucose in the cytoplasm is converted to pyruvate through a series of classic catalytic reactions; pyruvate does not enter mitochondria for oxidation but is directly reduced to lactate in a process dependent on lactate dehydrogenase (LDH)^23^. The accumulation of lactate in the human body is more dangerous than the accumulation of other molecular fuels, and a rise in serum lactate can lead to lactic acidosis^24^; therefore, lactate needs to be rapidly metabolically removed from tissues and circulation. Irreversible lactate removal is achieved by pyruvate dehydrogenase (PDH)^25^ (Fig. 1), which catalyzes the formation of pyruvate, which enters the TCA cycle in the form of acetyl-CoA^26^. Upon entry into the TCA cycle, acetyl-CoA forms a two-carbon unit because in mammals it cannot be converted into a three-carbon unit. Hence, the systemic balance between glycolysis and PDH flux may be a key determinant of lactate levels. PDH is a component of a catalytically active complex that is regulated by the phosphorylation status of the E1α subunit and NADH, which together inhibit PDH activity, resulting in elevated levels of circulating lactate under conditions of impaired mitochondrial activity or respiration^27^. In addition, lactate accumulation can activate gluconeogenesis in liver and skeletal muscle cells, through which lactate is converted to glucose and released into the blood to drive additional glucose consumption during energy expenditure^28^.

In addition to glycolysis, glutamine catabolism is another source of lactate in cancer cells^29^. Under the regulation of c-Myc, glutamine crosses the cell membrane through the amino acid transporter type 2 (ASCT2) and sodium-coupled neutral amino acid transporter 5 (SN2), enters the cytoplasm, and is converted to glutamate by glutaminase (GLS/GLS2). Then, glutamate is converted to α-ketoglutarate (α-KG) by glutamate dehydrogenase (GLUD) or a group of transaminases, including glutamate-oxaloacetate transaminase (GOT), glutamate-pyruvate transaminase (GPT), and phosphoserine aminotransferase (PSAT); α-KG then enters the TCA cycle. In this cycle, glutamine-derived carbon is converted to oxaloacetate, which is then converted to malate and leaves the mitochondria for subsequent conversion to NADPH and pyruvate by malic enzyme (ME1) in the cytoplasm. NADPH is required for the synthesis of fatty acids and sterols and for antioxidant mechanisms, while pyruvate is a source of lactate. Through this metabolic pathway, glutamine provides the carbon skeleton for lactate production and is a secondary source for lactate production in cancer cells.

MCTs constitute a class of transmembrane lactate transporters in the solute carrier family 16 (SLC16) family^7^. Among the 14 identified MCTs, MCT1-4 are expressed in a variety of tissues and are involved in catalytic proton coupling and the bidirectional transport of monocarboxylic acid^30^. MCT1 is an important subtype that was first discovered to be widely distributed in cells and to contribute to basal homeostatic maintenance. Under physiological conditions, synergistic activity of MCT1-4 promotes lactate shuttling between glycolytic and oxidizing cells, a key factor in lactate homeostasis within different tissues. In normal tissues, the high-affinity MCT1 maintains lactate homeostasis because it is responsible for the transfer of lactate according to the transmembrane lactate gradient^30^. Cells with high intracellular lactate concentrations, such as tumor cells, rely on the low-affinity MCT4 for lactate transport^30^. The transport process begins with the binding of free protons to MCT, followed by the binding of lactate, which undergoes a conformational change within the transporter and then is expelled on the other side of the membrane. The release of protons follows the release of lactate. When MCT is deprotonated, it undergoes a conformational change, restoring its initial structure in anticipation of the next transfer. Abnormal expression or inactivation of MCT1 has been associated with a variety of diseases, including symptomatic deficiency in lactate transport (SDLT), hyperinsulinemic hypoglycemia familial 7 (HHF7), and monocarboxylate transporter 1 deficiency (MCT1D). More importantly, high expression of MCT1, MCT2, and MCT4 is closely related to the development of cancer. Lactate shuttle mediated by MCT1 and other subtypes establishes intracellular connections and is involved in the synergistic metabolism between glycolytic tumor cells and oxidative tumor cells, thereby promoting tumor occurrence and development.

**Fig. 2:**
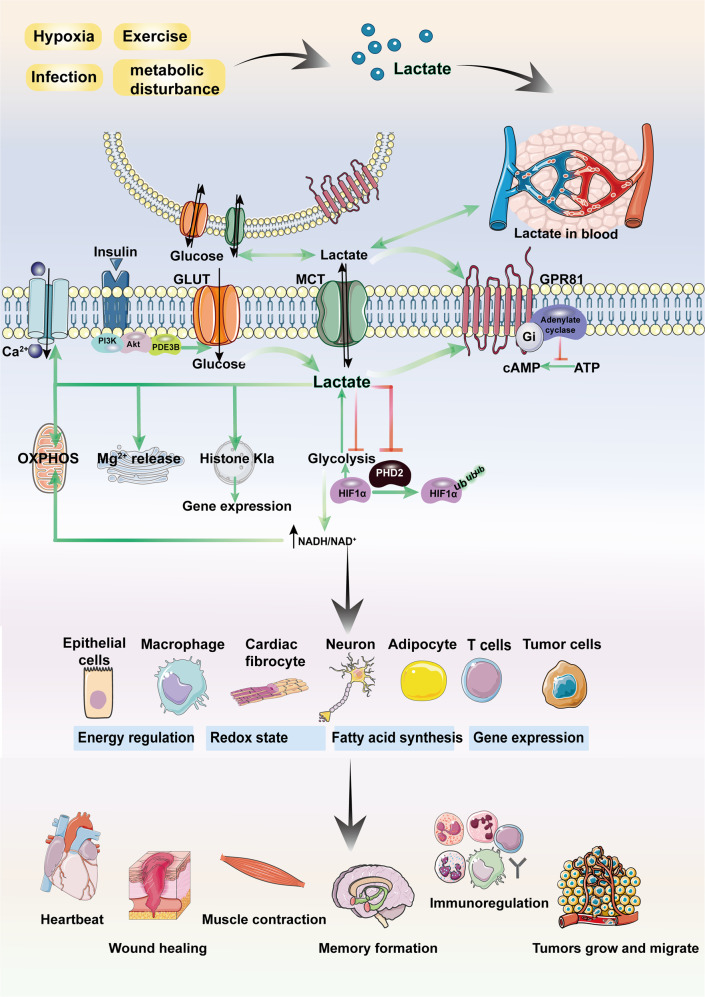
Lactate is involved in the regulation of cellular physiological and pathological processes. In addition to the intracellular production of lactate, lactate can enter target cells through intercellular shuttling involving nonchannel pathways or MCT1. As a signaling molecule or metabolic substrate, lactate is involved in glucose metabolism, fatty acid synthesis, redox homeostasis, and the PTM of proteins. Meanwhile, as a GPR81 ligand, lactate stimulates the GPR81 signaling pathway. Lactate has been shown to regulate muscle contraction, wound healing, memory formation, and tumor development. MCT monocarboxylate transporter; OXPHOS oxidative phosphorylation; GLUT glucose transporter. (Figure was partly created with SMART – Servier Medical ART)

A class of G protein-coupled receptors (GPRs) on the cell membrane interact with endogenous ligands to function as intermediate metabolites in hydroxycarboxylic acid generation during cellular energy metabolism. Among these GPRs, the lactate receptor GPR81 (Fig. 2) is highly expressed in adipose tissue, the kidney, skeletal muscle, the central nervous system, the heart and other organs and tissues^6^. Studies have found that GPR81 mediates biological processes such as lactate-induced energy metabolism, lipodieresis, neuronal protection, and inflammatory regulation^31–34^.

### Roles of lactic acid in biological processes

#### Energy regulation

Organisms can obtain energy from glucose through OXPHOS and glycolysis, both of which begin when glucose is broken down into two pyruvate molecules. Pyruvate enters mitochondria to participate in the TCA cycle during OXPHOS but is directly reduced to lactate during glycolysis. In the presence of oxygen, the production of each molecule of glucose through OXPHOS in the mitochondrial electron transport chain (ETC) leads to the rapid release of 36 or 38 molecules of ATP, but in the absence of oxygen, electron transfer cannot occur in mitochondria, and therefore, glycolysis becomes the only available pathway for glucose production^35^. The traditional view suggests that glucose consumption involves many more processes than lactate consumption and that lactate is a minor byproduct of rapid energy production under anaerobic conditions that is valuable only as a substrate for glucose production in the context of gluconeogenesis^36^ (Fig. 1). However, advances in research have led lactate to be reconsidered; it is no longer labeled a waste product but rather is recognized as a participant in glucose metabolism. Glucose has been determined to be the primary energy source in the brain, and circulating lactate is a supplementary source of glucose that satisfies excitatory brain activities when blood glucose levels are insufficient^37^. A study in 1988 reported that in the absence of glucose, lactate supported synaptic transmission in brain slices^38^. Other studies have shown that lactate can directly support neuronal activity: when lactate shuttling in hypothalamic ependymal-glial cells was inhibited, the energy balance in proopiomelanocortin (POMC) neurons was destroyed. Therefore, lactate, not glucose, is required to maintain POMC neuron activity^39^, which supports the energy balance in the whole body^40^. Lactate is largely a promoter of the TCA cycle. The concentration of lactate in circulation is higher than that of other energy substances; in mice, the level of circulating lactate was found to be 1.1-fold higher than that of glucose, and in fasting mice, the difference increased to 2.5-fold. A quantitative analysis showed that lactate has direct functions in organs, except the brain, of fasting mice, whereas glucose mainly contributes indirectly to TCA cycle metabolism. Lactate is the main fuel for the TCA cycle and is crucial for energy generation^41^, and studies have shown that lactate contributes more than glucose to the TCA cycle in lung and pancreatic cancer. Lactate also regulates energy based on the accumulation of both exogenous and endogenous lactate, which inhibits glycolysis through a product feedback pathway^42^. In addition, lactate has been found to activate Mg^2+^ release from the endoplasmic reticulum (ER) in a variety of cells, and this Mg^2+^ transport mechanism is linked to the main metabolic feedback loop and mitochondrial bioenergetic function to promote mitochondrial Mg^2+^ production^4^ (Fig. 2).

#### Redox buffer

As an important metabolic substrate, lactate is an intercellular and inter-tissue redox signaling molecule that provides energy for oxidative metabolism in many tissues and helps maintain redox homeostasis and tissue and whole-organism integrity. Energy metabolism is based on continuous redox reactions^43^. Oxidation leads to the release of electrons that are accepted by oxidized nicotinamide adenine dinucleotides (NAD^+^ or NADP^+^), which are then reduced to NADH or NADPH^44^; these reduced coenzymes release electrons during reoxidation through mitochondrial respiration or lactate fermentation, thus maintaining redox homeostasis in cells. In general, disruptions in homeostasis are detrimental to the body, regardless of whether the change leads to excessive oxidation or reduction. For example, when the NAD^+^/NADH (or NADP^+^/NADPH) ratio increases, cells enter an oxidation state, produce more active substances, undergo accelerating aging, and potentially cause cardiovascular disease; in contrast, in a high-reduction state, cells can accept electrons, their capacity to prevent oxidative stress is greatly reduced, and glycolysis is inhibited. Cytoplasmic LDH and mitochondrial ETC complex I are the main drivers of NADH oxidation to NAD^+^. First, the production and removal of lactate, as a metabolic intermediate, maintain electron flux through a specific process in which NADH is oxidized to NAD^+^ and H^+^, accompanied by LDH-mediated catalysis of lactate to pyruvate^45^. High concentrations of lactate have been shown to increase the NADH/NAD^+^ ratio, leading to the inhibition of GAPDH and PGDH activity and, subsequently, glycolysis and mitochondrial respiration^46,47^. Second, as a regulator of mitochondrial oxidative respiration, lactate controls the redox balance. When the demand for NAD^+^ in support of oxidation exceeds the rate of ATP turnover in cells undergoing active aerobic glycolysis, NAD^+^ is regenerated under conditions of limited mitochondrial respiration. At this point, cells tend to undergo glycolysis, which can lead to an increased NAD^+^/NADH ratio, and in response, PDH activation increases pyruvate oxidation, which can attenuate lactate accumulation and reduce the NAD^+^/NADH ratio^48^. When the mitochondrial ETC is dysfunctional, the intracellular NADH/NAD^+^ ratio is increased, and the lactate/pyruvate (L/P) ratio is increased in a reactive manner. When lactate oxidase (LOX) and catalase (CAT) irreversibly convert extracellular lactate into pyruvate in a timely manner, the intracellular NADH/NAD^+^ ratio tends to normalize, and ATP production increases^49^. Reactive oxygen species (ROS) are produced by the mitochondrial ETC^50^. Mitochondrial stress leads to increased ROS production, driving cells into a high oxidation state^51^. Data have confirmed that when lactate is actively oxidized, many ROS are produced in mitochondria, and excess ROS may lead to oxidative damage that can severely and irreversibly harm cells if not neutralized in a timely fashion^52,53^. A recent study found that the increased intake of lactate by neurons promoted ROS production, enhanced mitochondrial energy metabolism, and produced an oxidative state in neurons. Oxidative stress impairs ATP synthesis in mitochondria, resulting in higher ROS production; this vicious cycle ultimately leads to axon degeneration in the peripheral nervous system^54^. In addition, the intercellular transport of lactate is critical for the maintenance of the redox state. The inhibition of MCT1 and MCT4 activity and the continued lactate efflux result in intracellular acidification that inhibits LDH activity, leading to a greater loss in NAD^+^ regeneration capacity, ATP depletion, and ultimately cell death^55^. In conclusion, lactate is a redox buffer that contributes greatly to the oxidation state. However, when the ratio of oxidizing coenzyme to reducing coenzyme is unbalanced, lactate responds by regulating other forms of energy metabolism to stabilize the redox state of cells.

#### Regulator of fatty acid metabolism

Fatty acid anabolism is essential for cell membrane structure and function, energy storage, and signal transduction^56^. Lactate has been reported to accumulate at high concentrations, ranging from the physiological concentration of 1.5–3 to 10–40 mM, in inflammatory environments^57^. Lactate accumulation is known to promote fatty acid synthesis in cells, and lactate can replenish the intracellular pool of acetyl-CoA, which is necessary for fatty acid synthesis. Specifically, lactate has been reported to increase the activation of acetyl-CoA carboxylase (ACC), a key enzyme that regulates fatty acid synthesis, and to enhance the anabolism of fatty acids. Interestingly, Interestingly, lactate can induce CD4^+^ T cells to upregulate the expression of the lactate transporter SLC5A12, which mediates the uptake of lactate by CD4^+^ T cells, forming a positive feedback loop to increase the synthesis of fatty acids^58^. The lactate produced by glial cells can be transported into neurons, where it promotes adipogenesis by mediating ROS production^59^. A study on rat muscle showed that lactate underwent glyceroneogenesis, a seemingly unconventional way to promote fatty acid synthesis^60^. The fatty acid catabolism pathway involves β-oxidation, which releases a large amount of energy. Despite reports that prolonged exposure to lactate increased oleic acid oxidation^61^, the current understanding suggests that lactate inhibits fatty acid catabolism. A study of metabolic responses to exercise showed that the accumulation of circulating lactate during exercise was inversely related to fat oxidation^62^. Similarly, a study of post-acute sequelae of COVID-19 (PASC) found that patients with PASC presented with significant β-oxidation disorders and lactate accumulation in the blood during exercise^63^. Although the mechanism by which lactate regulates β-oxidation is currently unclear, it seems to be related to lactate signaling during exercise-induced mitochondrial adaptation. Some studies have shown that lactate production can be stimulated by treatment with high levels of lipids^64^, but further study is needed to determine whether a lactate feedback loop increases the inhibitory effect on fatty acid oxidation. Clearly, the effect of lactate on fatty acid oxidation is not unilateral. In the inflammatory stress response, acetyl-CoA produced by fatty acid oxidation can promote glycolysis through nonenzymatic acetylation, which can promote lactate formation^65^.

### Lactate shuttle

The lactate shuttle theory mainly describes intracellular and intercellular lactate shuttling, summarizing the entire process of lactate transmembrane migration^66^. As mentioned above, the complete removal of lactate through oxidation is achieved through the conversion to pyruvate by LDH, but the cellular location of this reaction remains unclear. The initiation of lactate oxidation to pyruvate is generally considered a cytoplasmic reaction, but to date, no evidence strongly supports this assumption. In fact, numerous studies have reported that contractions in skeletal muscle and beating heart muscle exponentially increase the lactate/pyruvate (L/P) ratio^67^. Considering this finding and a dynamic LDH model, Brooks proposed that lactate is shuttled within cells^68^. According to this theory, the lactate level increases more rapidly than the pyruvate level during exercise. Moreover, the oxidation of lactate to pyruvate does not seem to occur in the cytoplasm, where lactate is generated. In addition, lactate can enter mitochondria, where it undergoes direct oxidation without first being converted into pyruvate in the cytoplasm^68^. To reinforce this theory, Brooks proposed the mitochondrial lactate oxidation complex (mLOC) model, which involves MCT, its membrane partner basigin (BSG or CD147), LDH, and cytochrome oxidase (COX). According to this theory, the mLOC is located in the outer mitochondrial membrane and can oxidize lactate to pyruvate. However, this theory and model are controversial because no lactate transporter in this context has been identified and there is no evidence that LDH is activated in this context. In fact, one experimental study showed that mitochondria isolated from rat skeletal muscle failed to oxidize lactate and that LDH activity in mitochondria constituted only 0.7% of total cellular LDH activity^69^. However, another study found that MCT1, MCT2, and LDH colocalized with COX, a mitochondrial marker, in mouse cortical, hippocampal and thalamic neurons^70^. In another study, mitochondria isolated from the heart, skeletal muscle, and liver of rats were incubated with lactate and showed the ability to oxidize lactate^71^. Colocalization of MCT1, CD147, and LDH in the mitochondrial intima of L6 muscle cells provided evidence for the mLOC^72^. Hashimoto et al. summarized the following six points in support of the intracellular lactate shuttle theory. One, LDH was detected in mitochondria of the myocardium, liver and skeletal muscle of rats and humans by electron microscopy and laser scanning confocal microscopy. Two, the results of these analyses showed that mitochondria in the skeletal muscle and myocardium of rats and humans colocalized with MCT1. Three, LDH and MCT1, along with its molecular chaperone CD147, have been found in mitochondria isolated from rat and human skeletal muscle, myocardium, liver and kidney. Four, physiological experimental data showed that mitochondria oxidize lactate more quickly than pyruvate. Five, mitochondria in the myocardium and skeletal muscle cells of humans and other mammals can oxidize lactate, as indicated by isotope tracing and nuclear magnetic resonance imaging (MRI). Six, the results of mitochondrial proteomic studies support the lactate shuttle hypothesis^71^. The intracellular lactate shuttle theory subverts conventional thinking, to a certain extent, because it updates the theory of inherent lactate oxidation in cells. Furthermore, the lactate shuttle theory suggests that lactate production during exercise is an adaptive response by cell signaling molecules, explaining the mechanism through which training enhances lactate removal through oxidation.

The concept of intercellular lactate shuttling was proposed and systematically explained in 1985^2^. The theory suggests that at the beginning of exercise, lactate is rapidly produced and accumulates in muscle cells; then, some of this lactate enters tissues, where it is internalized and oxidized by adjacent cells, whereas the remaining lactate enters the blood circulatory system and is delivered to the heart, liver, and kidney, where it is a substrate for oxidative energy production and gluconeogenesis. Brooks and his team explained the important roles of lactate identified during the formation and validation of the lactate shuttle theory. They not only confirmed that aerobic oxidation is the main pathway of lactate removal during and after exercise but also emphasized the function of lactate as an energy source and a substrate for gluconeogenesis. More importantly, this theory recognizes a new biologically significant role of lactate by detailing how it regulates oxidation and intercellular signal communication^73–75^. With advances in research, lactate shuttling between skeletal muscle and the heart has been increasingly reported and verified to provide energy to the heart^76^. Lactate shuttling has also been identified between cardiomyocytes and fibroblasts. In a coculture system, the production of lactate increased in fibroblasts, and MCT1, which induces the influx of lactate, migrated to the myocardial membrane^77^. This study provided direct evidence that lactate is a paracrine signaling molecule. Another study showed that in the brain, lactate produced by astrocytes entered neurons, subsequently participated in energy metabolism, and was converted into pyruvate and acetyl-CoA to regulate fatty acid synthesis^59^. Defective lactate shuttling from glial cells to neurons led to dysregulated brain metabolism, causing degeneration similar to that in Alzheimer’s disease (AD)^78^. In line with these findings, disruption of lactate shuttling has been shown to negatively affect motor function and destabilize motor units^79^. In the kidney, lactate is produced by the proximal tubule and consumed by the distal tubule, and the lactate shuttle allows lactate to fulfill its function as a fuel through transfer from the proximal to the distal nephron^80^. In summary, the lactate shuttle theory is applicable to a variety of practical areas, such as sports nutrition and hydration, acidosis, the treatment of traumatic brain injury, the maintenance of blood glucose, the reduction of inflammation, cardiac support after heart failure, and myocardial infarction, and the enhancement of cognition, whereas dysregulation of the lactate shuttle disrupts metabolic flexibility and supports tumorigenesis^81^.

Lactate shuttling between different cell populations in the tumor microenvironment (TME) is a new phenomenon in the field of tumor biology. Lactate shuttling occurs in many physiological and pathological conditions, where in lactate is exported by one cell type and imported by another cell type. The well-known Cori cycle involves lactate shuttling between skeletal muscle and the liver. Because lactate is an energy-rich metabolite that can be used as a precursor for gluconeogenesis and ATP synthesis, it is especially important for this metabolite to shuttle through the TME, which contains hypoxic and normoxic cell populations. In essence, glycolysis is dependent on oxygen as cancer cells cannot oxidize lactatea, and glucose is known to decrease oxygen uptake; therefore, mitochondrial respiration is used for ATP synthesis. As the understanding of tumor cell heterogeneity based on oxygen availability has increased, there has been a paradigm shift in our current understanding of cancer related to the Warburg effect. Because tumors grow faster than blood vessels can form, cancer cells close to blood vessels receive oxygen and are therefore normoxic, while those farther from blood vessels lack sufficient oxygen supply and are hypoxic. Specifically, hypoxic tumor cells utilize LDH-A to produce lactate, which is exported from the cell to be absorbed by normoxic tumor cells, which convert it to pyruvate through LDH-B to produce ATP. According to the functional characteristics of MCT1 and MCT4 and the differential regulation of hypoxia-related genes, MCT4 mediates the release of lactate by hypoxic tumor cells, while MCT1 mediates the uptake of lactic acid by normoxic tumor cells, highlighting the mutual relationship and metabolic symbiosis between cancer cells in different parts of a single tumor. This metabolic symbiosis occurs between different types of cancer cells within the tumor and between normoxic cancer cells and tumor-associated stromal cells.

### Lactylation modification

Yingming Zhao at the University of Chicago used high-performance liquid chromatography (HPLC)–tandem mass spectrometry (MS/MS) to detect core histone proteins in human MCF-7 cells. They found that the mass shift on the lysine residues of three proteolytic peptides was the same as that caused by the addition of a lactyl group to the lysine ε-amino group^16^. This study demonstrated for the first time the presence of histone lysine lactylation (Kla) and indicated that Kla is a new type of epigenetic modification that occurs after the translation of lactate-derived proteins. Surprisingly, many studies have shown the accumulation of histone Kla on gene promoters in cells stimulated by hypoxia, interferon (IFN)-γ, lipopolysaccharide (LPS), or bacterial attack to produce lactate^16,82^, thereby directly regulating gene expression^16^ (Fig. 1).

To date, research on Kla has focused on both histone and nonhistone aspects. Histone Kla has unique time dynamics compared to those of histone acetylation. Histone Kla is significantly increased on the promoters of M2-like genes in the later stage of stimulated M1 macrophage polarization, suggesting that histone Kla probably acts as a lactate clock to promote the switch from an inflammatory phenotype to a steady-state phenotype in macrophages. This switch occurs in the later stages of inflammation, which may be related to wound healing. B-cell adapter for PI3K (BCAP), a signal adapter for Toll-like receptors (TLRs), has been shown to play a vital role as an internal cell switch in promoting macrophage gene expression that supports the transition from a proinflammatory state to a reparative state^83,84^. Ricardo et al. revealed that mice with macrophage-specific BCAP deletion had decreased Arg1 and Klf4 expression, failed to recover from dextran sodium sulfate-induced colitis, and eventually died^82^. Other studies found that BCAP deficiency also resulted in defective aerobic glycolysis and reduced lactate production, causing decreased histone Kla. The addition of exogenous sodium lactate (NaLa) to bone marrow-derived macrophages (BMDMs) lacking BCAP promoted histone Kla and recovered the decreases in ARG1 and Klf4 expression mediated by BACP deficiency^82^. These observations led to the conclusion that BCAP is an upstream adapter that connects TLR signals with the optimal aerobic glycolysis in macrophages, resulting in the lactate production necessary for proper histone Kla to promote the rescue of gene expression. This phenomenon thereby promotes the transformation of macrophages from a proinflammatory phenotype to a reparative phenotype. Another recent study found that lactate produced by probiotic *Saccharomyces cerevisiae* effectively inhibited BMDM activation and thus attenuated ulcerative colitis, as lactate increased H3K9 acetylation and H3K18 lactylation in BMDMs. Regarding, the specific mechanism for this therapeutic effect, the genes or proteins regulated by H3K18 lactylation may be involved in the inhibition of M1 macrophage polarization and the NLRP3 inflammasome^85^. In addition, increased lactate levels in human alveolar macrophages led to an increase in histone Kla^86^. Increased levels of histone Kla were observed in a mouse model of bleomycin-induced pulmonary fibrosis and in human pulmonary fibrosis. Results from CHIP assays using lactate-treated BMDMs confirmed increased histone Kla in the promoter regions of the ARG1, PDGFA, THBS1, and VEGFA genes, leading to their significant upregulation. It is obvious from these observations that lactate activates gene expression by inducing histone Kla of the promoters of profibrotic mediators.

AD was recently shown to be associated with histone lactylation^87^. Studies showed increased lactylation of H4K12 in AD mice, and this histone modification was enriched at the promoter of glycolysis-related genes and activated transcription, thereby increasing glycolytic activity. Finally, activation of the positive feedback cycle of glycolysis-H4K12La-PKM2 was shown to exacerbate microglial dysfunction in AD^87^.

**Fig. 3:**
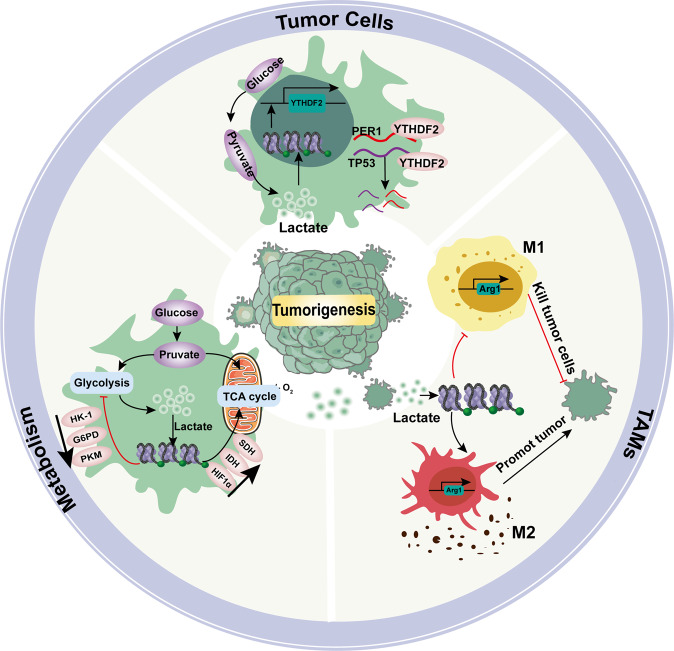
Mechanism by which lactylation promotes tumorigenesis. Lactylation leads to tumor immunosuppression by maintaining TAM homeostasis. Lactylation directly promotes the expression of the oncogene YTHDF2 in tumor cells. Lactylation maintains the metabolic homeostasis of tumor cells. Lactylation may lead to tumor immunosuppression and immune escape by inhibiting the function of various immune cells in the TME. HK-1 hexokinase-1; G6PD glucose-6-phosphate dehydrogenase; PKM pyruvate kinase; SDH succinate dehydrogenase; IDH isocitrate dehydrogenase; TCA cycle tricarboxylic acid cycle

Histone Kla has been proven to promote tumor development (Fig. 3). Yu et al. found that increased histone Kla was associated with the poor prognosis of patients with ocular melanoma^88^. Inhibition of histone Kla in ocular melanoma cells confirmed the significant positive correlation of ocular melanoma with intracellular histone Kla. The underlying mechanism leading to the occurrence of melanoma involves activating YTHDF2 expression by increasing histone Kla of the promoter. YTHDF2, a reader of m6A, plays a vital role in promoting tumorigenesis in ocular melanoma. Although some studies support the notion that histone Kla is essential for tumor growth, others state that histone Kla cannot transform normal cells into cancerous cells, indicating a role of histone Kla in promoting the growth of developing tumors rather than driving initial tumorigenesis. Tumor cells interact with extracellular matrix (ECM) components to form a complex TME. Hypoxia and deficiencies in blood-derived nutrients are the main characteristics of the TME, and tumor cells adjust their metabolism to survive in this unfavorable environment^89^. A subsequent study on non-small cell lung cancer (NSCLC) confirmed that histone Kla downregulated the gene expression of the glycolysis-related enzymes hexokinase (HK)-1 and pyruvate kinase (PKM) and upregulated that of the TCA cycle-related enzymes succinate dehydrogenase (SDH) and isocitrate dehydrogenase (IDH)^90^. These results reveal that lactate-mediated changes in metabolic gene expression via histone Kla induce glucose uptake by tumor cells; therefore, lactate plays an essential role in the metabolic disorders of NSCLC. Similarly, the study by Zhao et al. verified that ARG1 gene expression was positively correlated with histone Kla in TAMs isolated from B16F10 melanoma and LLC1 lung tumor tissues^16^. In addition, histone Kla has been detected in various other tumor cell lines, such as HeLa cells, MCF-7 cells, and HepG2 cells (Table 1); these data support further investigation into the mechanisms by which histone Kla is regulated in these cells. In summary, lactate in the TME induces immunosuppression and promotes immune evasion in tumors, thereby helping maintain tumor survival and growth. Moreover, histone Kla fulfills the goals of lactate-mediated signaling to support tumor growth. Additionally, histone Kla regulates the transcription of tumor-related genes such as YTHDF2, which promotes tumor growth, metastasis, and invasion by encouraging the degradation of downstream tumor suppressors. Histone Kla induces the expression of TCA cycle-related enzymes such as SDH, which promotes tumor cell metabolism and growth. The genetic and phenotypic heterogeneity of tumor cells is a major obstacle to cancer treatment. Random genetic changes create highly chaotic and unpredictable intratumoral heterogeneity. Hence, the regulation of histone Kla could be a powerful target for adjuvant treatment to influence the epigenetic landscape of tumors.

**Table 1:** The regulation of lactylation in cell lines and diseases

Cell lines	Lactylated protein(s)/site(s)	Function and mechanism	Disease
MCF-7	H3K9,18,23,27,56,122	N/A	Breast cancer16
	H4K5,8,12,31,77,91		
HeLa	H3K9,18,23,27,79	N/A	Cervical cancer16
	H4K5,8,12,16,31,77,91		
	H2AK11,13,115		
	H2BK5,11,15,16,20,23,43,85,108,116,120		
MEF	N/A	N/A	N/A
HCT116	H3K18	N/A	Colon cancer16
BMDM	H3K14,18,23,27,56	Wound healing 16; inflammation repair 70; tumor cell proliferation; pulmonary fibrosis 72; upregulate ARG1, PDGFA, THBS1, and VEGFA.	N/A
	H4K8,12,31,91		
	H2AK11,115		
	H2BK5,11,15,16,20,85,108		
HepG2	H3K18	N/A	Hepatocellular carcinoma16
HEK293T	H3K18	N/A	N/A
LLC1	H3K18	N/A	Lung cancer 16
B16F10	H3K18	N/A	Cutaneous melanoma16
PIG1	H3K18	N/A	N/A
OCM1/OMM1/MUM2B/CRMM1/2/CM2005.1	H3K18	Tumorigenesis 74; upregulate the oncogene YTHDF2.	Ocular melanoma74
BEAS-2B/A549/H1299	H4	Tumor cell proliferation 76; upregulate SDH and IDH; downregulate HK-1 and PKM.	Non-small cell lung cancer76

Notes: *N/A* not available

Kla has been found to also occur on nonhistone proteins. In terms of acute inflammation, abnormally elevated levels of lactate in the blood of sepsis patients can be taken up by macrophages, thereby increasing the Kla of intracellular HMGB1 protein^91^. This research also proved that lactate can mediate the transfer of HMGB1 from the nucleus to the cytoplasm through GPR81 and MCTs and can cause an increase in HMGB1 modification by lactylation. Meanwhile, lactylated HMGB1 is secreted and released through the exosome pathway, whereby it damages endothelial integrity and increases vascular permeability, leading to endothelial barrier dysfunction and promoting the development of sepsis. A recent study revealed that lactate accumulated in the TME regulated the N6-methyladenosine (m6A) modification of tumor-infiltrating myeloid cells (TIMs) mediated by the RNA methyltransferase METTL3 through Kla to promote the immunosuppressive function of TIMs and mediate tumor immune escape^92^. Moreover, lactate promoted METTL3 transcription in TIMs through histone Kla. In addition, Kla can occur in the zinc finger domain (ZFD) of METTL3, which functions as the target recognition domain (TRD), thereby enhancing METTL3 binding and catalysis of the m6A modification of target RNA. Systemic lupus erythematosus (SLE) is an autoimmune inflammatory connective tissue disease. Defects in red blood cell (RBC) development have been reported as a possible trigger of SLE. During RBC maturation, the regulatory metabolic switch responsible for activating the ubiquitin proteasome system (UPS) is mediated by hypoxia-inducible factor (HIF). However, the UPS undergoes Kla in SLE^93^, which impacts UPS activation mediated by the metabolic switch, resulting in an inability to clear mitochondria by autophagy and a consequent increase in these organelles in mature RBCs. Once abnormal RBCs are taken up by macrophages, the mitochondrial DNA in the RBCs stimulates the powerful inflammatory cGAS/STING pathway, which promotes the production of type I IFN and causes SLE^93^. Recently, a study explained how lactate levels in the brain are regulated by systemic changes in brain cells, neural excitement, and behavior-related stimuli, thereby leading to Kla^94^. Murine brain neurons stimulated with electrical convulsions were excited, accompanied by an increase in Kla. In the social frustration stress model of depression with elevated brain lactate levels, the increase in brain neuron excitability also increased Kla. Studies have reported reduced social behavior and increased anxiety-like behavior in mice. It is hypothesized that stress-induced meta-excitation may lead to Kla, which affects emotion-related behaviors in such cases. The report mentioned above was from a pioneering study that led to the discovery of the potential role of Kla in neuronal activity.

Classical acyltransferases such as p300/CREB binding protein (CBP) are known to catalyze various acylation modifications, including the acetylation of transcription factors, histones, and other nuclear proteins, thereby regulating gene expression^95–97^. In vitro cell-free experiments showed that p300 may catalyze the chemical Kla reaction, with a strong dependence on the p53 pathway. p300/CBP has been confirmed by several studies to regulate histone lactylation in macrophages and induced pluripotent stem cells (iPSCs)^16,86,91,98,99^. In addition, there are reports that p300 can serve as a “writer” of lactylation on the YTHDF2 promoter in ocular melanoma cells^88^. Another major study that led to the discovery of histone Kla demonstrated that the addition and removal of this modification were enzymatic processes driven by HDACs. A detailed analysis of the mechanism of delactylation was carried out in vitro using 18 recombinant HDACs and core histones as substrates; the results revealed that HDAC1-3 and SIRT1-3 reduced histone (including H3K18 and H4K5) Kla and that HDAC3 was the most potent eraser of Kla^99^.

Consistent with many other PTMs, Kla is theoretically regulated by adding and removing lactyl groups from histone proteins^100,101^. However, the currently understood biochemical process of lactylation may rely on the function of two metabolic mechanisms. Among the components of these mechanisms, lactyl-CoA is closely associated with enzymatic lactylation, and lactyl-glutathione (LGSH) participates in nonenzymatic lactylation (Fig. 1)^102^. Studies have shown that methylglyoxal (MGO) is a byproduct of glucose metabolism; in glycolysis, MGO is generated to produce triose phosphate and glyceraldehyde triphosphate through spontaneous nonenzymatic dephosphorylation^103^. Under physiological conditions, glyoxalase (GLO) activity maintains a low level of MGO. GLO1 promotes the synthesis of glutathione (GSH) and MGO to produce D-LGSH, and GLO2 hydrolyzes LGSH to produce GSH and D-lactate^104^. A previous study found an increase in Kla accompanied by a significant increase in LGSH in GLO2-knockout cell lines^102^. This study further revealed that proteins that underwent Kla were enriched in carbon metabolism and glycolytic pathways. Taken together, these data indicate that glycolysis-related proteins undergo nonenzymatic Kla regulated by glucose metabolites.

## Lactate and allosteric binding

Lactate is commonly produced as three isomers, D-lactate, L-lactate, and racemic DL-lactate, because of carbon atom asymmetry. L-Lactate is the main form in the human body, and LDH-A reduces pyruvate to lactate. Studies showed that excessive lactate accumulation in the cytoplasm of HepG2 hepatoma cells exposed LDH-A to an acidic environment that induced an allosteric transformation resulting in reduced activity^105^.

In contrast, D-lactate is a primary metabolite in gut bacteria^106^, and only approximately 1–5% of L-lactate content is derived from pyruvate metabolism^107^. Generally, D-LDH is not present in mammals, and D-lactate is metabolized by D-α-hydroxy acid dehydrogenase, which is active in only a very narrow pH range and induces very slow catalysis. Therefore, most scholars believe that D-lactate content in humans under normal conditions is too low to activate enzymes related to catabolism^108^. However, recent studies have suggested that D-LDH is expressed in human and mammalian mitochondria^109^. Moreover, D-lactate has been shown to be metabolized more readily than initially thought, as confirmed by studies on the half-life of D-lactate in plasma and its excretion in urine after infusion or oral administration^110,111^. D-Lactate may be involved in the transport of metabolic substrates in vivo. Studies have reported the identification of three new D-lactate/H^+^ cotransporters, D-lactate/pyruvate reverse transporters, and D-lactate/malate reverse transporters that transport D-pyruvate from the cytoplasm to the mitochondrial membrane. Hence, D-lactate/malate reverse transporters are suspected to localize to the mitochondrial intima where D-pyruvate is transported after mitochondrial D-LDH-mediated oxidation, and malic acid is transported in the reverse direction to the cytoplasm^108^.

## Roles of lactic acid in pathophysiological processes

### Inflammatory responses

**Fig. 4:**
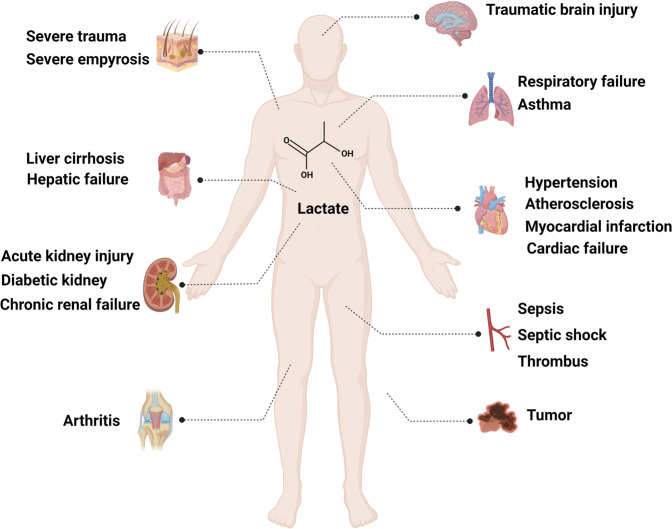
Lactate contributes to various diseases. Lactate is involved in the regulation of cardiovascular system, respiratory system, digestive system, urinary system, and other diseases. Lactate plays an important role in clinical diagnosis and prognosis of diseases. (Figure was created with Biorender.com)

Inflammatory response involves a variety of acute and chronic diseases in almost all organs^112–116^. In addition to participating in inflammatory injury and immune energy metabolism, accumulated lactate triggers the activation of a series of cellular signaling pathways that regulate inflammatory progression and tumor immune tolerance. Notably, these regulatory effects are not related to the ability of lactate to acidify the cellular environment. The occurrence of acute inflammation is generally considered a host defense mechanism, but the unrestrained activation of acute inflammation will lead to tissue necrosis and prolonged disease. Recent studies have confirmed that lactate has an inhibitory effect on acute inflammation (Fig. 4).

NF-κB Signaling regulates genes involved in numerous biological processes such as innate and acquired immunity, inflammation, stress response, B cell formation, and lymphoid organ formation^117,118^. In the classical pathway, NF-κB/Rel binds to and is inhibited by IκB^119^. Proinflammatory factors, LPS, growth factors, and antigen receptors activate the IKK complex (including IKKβ, IKKα, and NEMO), and the latter phosphorylates the IκB protein, leading to ubiquitination and lysosomal degradation of IκB, resulting in the release of NF-κB. Activated NF-κB is further phosphorylated and transferred into nuclear-induced target gene expression. TLRs Pathway also plays a significant role in inflammation. TLRs signal transduction from the intracellular TIR domain of the receptor (Toll/IL-1 receptor) When stimulated by ligands, MyD88 binds the IRAK(IL-1 receptor-associated kinase) to TLRs through the interaction of the two molecular death domains^120^. IRAK-1 is phosphorylated and activated, which then binds to TRAF6, resulting in the activation of JNK and NF-KB^121^. Lactate was shown to inhibit the production of inflammatory cytokines and the degranulation of mast cells in vitro^122^, delay the LPS-induced upregulation of monocyte inflammatory genes, and reduce nuclear NF-κB accumulation. Moreover, lactate was shown to significantly reduce the production of TNF-α and IL-6 and the activation and nuclear translocation of NF-κB and YAP through the GPR81 pathway in LPS-stimulated macrophages^123,124^. Lactate can inhibit TLR-mediated activation of mononuclear macrophages, delay the phosphorylation of Akt and the degradation of IκBα, and inhibit the secretion of cytokines TNF-α, IL-23, and chemokine CCL2, CCL7^123^.

Macrophages undergo polarization into different phenotypes based on local microenvironmental stimuli. As mentioned earlier, the activation of glycolytic pathways is accompanied by the activation of hypoxia and inflammation, which increase the production and release of lactate^125^. Macrophages must react quickly to synthesize and release a burst of proinflammatory factors to fight pathogenic microorganisms and simultaneously recruit more immune cells to the inflammation site to cope with bacterial infections. Such phenomena result in the M1 polarization of macrophages. During this process, macrophages secrete various proinflammatory cytokines (e.g., TNFα, IFNγ, IL-12, etc.), shifting the metabolic pattern toward aerobic glycolysis^126–128^. In contrast, M2-polarized macrophages express and release more anti-inflammatory factors (e.g., ARG1, TGFβ, etc.), which are generally involved in tissue repair and wound healing^129^, and in the late stage of inflammation, macrophages were often observed to have an M2-like immunophenotype which played a key role in the pathogenesis of immune system dysfunctions^130^. High concentration of lactate infiltration has a great impact on the polarization and cell function of monocyte-macrophages. First of all, lactate can inhibit the key glycolysis enzyme PFK-1 and promote the decomposition of active PFK-1 into less active dimer, thus reducing the glycolysis flux of monocytes^131^ and affect the immune function and further differentiation of monocytes. Secondly, lactate can be used as a signaling molecule to induce polarization of M2-like macrophages^132^. Selleri et al. reported that lactate secreted by human mesenchymal stromal cells induced the differentiation of monocytes into M2 macrophages in a dose-dependent manner^133^.

Mast cells are unique tissue-resident immune cells of the myeloid lineage that have long been implicated in the pathogenesis of allergic and autoimmune disorders^134^. Lactate was found to target MAS-associated G protein-coupled receptor X2 (MRGPRX2) expressed by mast cells to inhibit both the early (calcium mobilization and degranulation) and late (chemokine/cytokine release) phases of mast cell activation in asthma^135^. In addition, lactate has been shown to rely on MCT1 to inhibit the production and degranulation of inflammatory cytokines in IgE-mediated mast cells, thereby limiting mast cell-mediated inflammation^122^.

Studies in mouse models of colitis showed that activation of the lactate receptor GPR81 reduced inflammation^136^, In a DSS-induced mouse model of colitis, MCTs-mediated increased uptake of lactate inhibits overactivation of inflammasome NLRP3 and its downstream caspase-1 pathways in macrophages^85^. In septic acute kidney injury, the lactate-activated PD-1/PD-L1 pathway induced immunosuppression by evoking lymphocyte apoptosis^137,138^. In addition, lactate inhibits the activation of inflammasomes that cause liver and pancreatic damage through the GPR81 pathway^139^. In sepsis, accumulated lactate can inhibit the activation of NF-KB pathway, the production of inflammatory factors, the glycolysis process and ATP production through MCT1 in mast cell, and participate in the inflammatory stage to the secondary immunosuppression stage of sepsis^140^. Thus, local administration of lactate can be one of the potential treatments for acute inflammatory diseases.

Chronic inflammation is characterized by greater infiltration of T lymphocytes and macrophages and, in contrast to acute inflammation, is enhanced by lactate^141^. Lactate has been reported to inhibit the migration of T cells and thus retain T cells at the site of inflammation, thereby prolonging chronic inflammation by increasing the production of inflammatory cytokines and decreasing cell lysis^142,143^. In mouse arthritis models, lactate activated the expression of its transporter SLC5A12, which mediates the entry of lactate into CD4^+^ T cells, and promoted IL-17 production through the PKM2/STAT3 signaling pathway. Conversely, blocking SLC5A12 was shown to decrease disease severity^58^. Additionally, studies have shown that LDH-A is overexpressed in all CD8^+^ T-cell subsets in the context of rheumatoid arthritis, and LDH-A inhibition can alleviate the inflammatory and destructive effects of CD8^+^ T cells in the development of autoimmune diseases^144^; to some extent, these findings may provide indirect evidence that lactic acid promotes chronic inflammation. Enhanced glycolysis causes the localized accumulation of lactate in the fibrotic lung, which promotes the profibrotic activity of alveolar macrophages^145^. Studies have also found that lactate is an effective stimulant of the profibrotic phenotype of macrophages^146^.

The TME is hypoxic, and both hypoxia and high lactate concentrations may be key drivers of the recruitment and polarization of tumor-associated macrophages (TAMs)^147^. In recent years, tumor immunity has attracted increasing attention. Immunosuppression plays an important role in tumor growth and invasion. Lactate plays a crucial role in regulating the functions of macrophages and lymphocytes in the process of immune suppression.

Hypoxic tissues secrete high levels of the chemokines, HIF1/2 and endothelin-2, which attract macrophages to hypoxic areas to affect the local immune response. Continuous tumor antigen stimulation and immune activation prompt a state of exhaustion or remodeling in the immune effector cells in the TME, rendering these cells unable to perform their normal functions. TAMs integrate hypoxia and lactate levels into activation of the MAPK signaling cascade to promote malignant and tumorigenic factors, such as the expression of arginase 1 (ARG1) and mannose receptor type C1 (MRC1)^148,149^. M1-like macrophages in the TME inhibit tumor cell growth. M1 macrophage polarization is positively correlated with a favorable clinical prognosis in many types of cancer, while M2-polarized macrophages promote tumor occurrence and development^150–154^. Macrophages in an acidic TME tend to have an M2 phenotype: tumor-derived lactate induces M2 macrophage polarization by activating the ERK/STAT3 signaling pathway. Inhibition of ERK/STAT3 signaling is known to hinder tumor growth and angiogenesis by restraining lactate-mediated M2 macrophage polarization^155^. Previous studies have shown that the treatment of BMDMs with lactate extracted from tumor cells drives an M2-like phenotype characteristic of TAMs^156^. In summary, lactate in the TME induces immunosuppression and promotes tumor immune evasion, which helps maintain tumor growth and survival.

Inflammatory factors in the cellular microenvironment activate the immune response, thereby promoting glycolysis and increasing the production and release of lactate^157^. The response to lactate by different cells is highly variable. For example, immune cells in cancer respond to lactate in the opposite way as those in chronic inflammatory diseases, although both sets of cells ultimately promote the disease. Moreover, tumor-derived lactate has been reported to inhibit the immune response to the tumor itself and to promote the expression of anti-inflammatory genes, thus creating a conducive microenvironment for tumor growth^158,159^. Although the significant effect of lactate on cellular function is well established, its precise contributions remain to be further explored^13^.

It is not difficult to find interesting effects of lactate signaling. As an immune system modulator, lactate seems to have contradictory potential proinflammatory and anti-inflammatory functions. For example, lactate signaling inhibited the LPS-induced expression of a series of cytokines and chemokines, as measured in cell culture medium^160^. In macrophages and monocytes, lactate inhibited glycolysis and various specific receptor signaling cascades^123^. Lactate can also regulate and control gene expression through PTMs; therefore, lactate not only inhibits inflammatory macrophage (M1) function but also enhances the regulation of anti-inflammatory M2 polarization, helping reduce macrophage-mediated inflammation and restore homeostasis through an intrinsic regulatory feedback pathway. In contrast, lactate has been shown to enhance the macrophage secretion of IL-6, matrix metalloproteinase 1 (MMP1), and IL-1β and to increase NF-κB activity through MCTs^161^. In addition, lactate appears to play an inconsistent role in T cells. Lactate was shown to inhibit the proliferation, degranulation, and active cytolysis of CD8^+^ T cells and to release inflammatory mediators of CD8^+^ T cells^162^. However, in the CD4^+^ T-cell lineage, lactate can increase the release of IL-2^143^, inhibit the inflammatory function of regulatory T cells (Tregs), and promote the differentiation of T-helper 17 (Th17) cells^163^. There are three possible explanations for the different regulatory effects of lactate in immune cells. First, the microenvironment of immune cells differs from that of other somatic cells; for example, granulocyte macrophage colony-stimulating factor (GM-CSF) can induce an increase in M1 and regulatory M2 mediators in monocytes in the presence of lactate, and this induction is consistent with the phenotype acquired by TAMs^164^. Second, the expression of lactate transporters in effector T cells results in different functions of lactate; for example, the differential effects in CD8^+^ and CD4^+^ T cells are mediated by the selective expression of MCT-1 and SCL5A12, respectively^143^. Third, the concentration of lactate and its effect on culture medium acidity are related to different effects; for example, 12.5 mM lactate reduced the pH of the culture medium to 6.7 and inhibited cytokine production^140,165^, and adjusting the pH to 7.4 reversed the inhibitory effect of lactate in dendritic cells^166^. Needless to say, lactate can indeed be used as a research target for clinical diagnosis and treatment of inflammatory diseases. At present, the determination of serum lactate is of great significance for clinical monitoring of the course of diseases such as acute peritonitis, acute pancreatitis, sepsis, and septic shock. At the same time, clinical trials have confirmed that serum lactate concentration can be used to guide the medication of clinical patients, as the goal of treatment^167,168^, prognosis forecast^169,170^, and predict the prognosis^171–174^. Experiments have revealed that lactate can prevent cardiac dysfunction in rats with sepsis, improve microcirculation, and reduce inflammation^175^.

### Memory formation and neuroprotection

At rest, the brain is considered a net lactate producer^176,177^. The lactate produced by the cells in the brain is released to the extracellular fluid gap through the membrane, and then the blood vessels or lymphatic system within the gap is led into the blood, thus entering the systemic circulation. When the blood lactate level rises, the brain transforms into a net organ of lactate uptake, and blood lactate is transported into the center by MCT on the blood-brain barrier. The uptake of lactate by brain cells increases, and the brain can remove up to 11% of the body lactate in exercise state^176^.

Under different physiological and pathophysiological conditions, lactate exerts different effects at the molecular and organ levels in the brain to influence behaviors, such as facilitating learning and memory and enabling the regulation of emotions. Lactate and MCT-mediated lactate transport have been shown to be important for brain energy metabolism^178^. Pyruvate could not rescue memory impairment caused by MCT2 downregulation in neurons, but an increase in endogenous lactate levels increased learning-induced mRNA translation and Arc/Arg3.1 expression, suggesting that lactate plays a key role as fuel for the neuronal responses required for long-term memory^179^. N-Methyl-D-aspartate receptors (NMDARs) are glutamate receptors and typical mediators of synaptic plasticity. Lactate enhances the NMDAR-dependent inward current flow and calcium influx induced by glutamate and glycine, thereby activating NMDARs and downstream ERK1/2 signaling and increasing the expression of c-Fos and Zif268, which are involved in neuronal plasticity and activity maintenance^180,181^. The abovementioned evidence for lactate shuttling between astrocytes and neurons suggests that lactate homeostasis plays a coordinating role in long-term memory formation. Disrupted gene expression of the lactate transporter MCT1 in brain endothelial cells eliminated lactate transport and impaired hippocampal neurogenesis and cognitive function^182^. Lactate injections prevented the destruction of memory retention caused by damage to the hippocampus, but injections of glucose at the same concentration did not have the same effect^183^. Studies have shown that the lactate content in the cerebral cortex and hippocampus decreases during memory impairment in mouse models of AD^184^. Coincidentally, exercise-generated lactate enters the hippocampus through the lactate shuttle and increases the expression of brain-derived neurotrophic factor (BDNF) by activating SIRT1^185^, and increases in BDNF expression improve cognition by facilitating learning and memory formation. Moreover, lactate signaling between astrocytes and neurons is disrupted in AD, amyotrophic lateral sclerosis (ALS), and schizophrenia^180^.

In conclusion, lactate not only acts as an energy substrate in the brain, but also plays a certain role in the maintenance of long-term memory formation and cognitive function. In addition, lactate can be used as a signal molecule to bind to GPR81 receptor in the brain^186^ for reducing excitatory injury, which suggests that lactate may be involved in the whole brain metabolism and functional regulation (Table 2).

**Table 2:** Summary of lactate in disease and the related signaling pathways

Disease	Mechanism	Clinical significance
Cancer	Energy metabolism	Potential therapeutic targets
	PD-L1/PD-1 pathway T-cell apoptosis	
	PAR-VEG/VEGFR2	
	Histone/Non-Histone lactylation	
	Activate the ERK–STAT3 pathway, GPR132 and Notch	
	Stabilize the HIF1 pathway	
	Inhibit NFAT, NKp46, and mTOR signaling	
Traumatic brain injury	Energy metabolism	Neuroprotective effects
		Biomarker of systemic physiology
		Therapy for treating encephaledema
Cardiovascular disease	Energy metabolism	Biomarker of myocardial injury
	GPR81/KLF2-mediated down-regulation of inflammatory cytokines IL-6, IL-8, MCP-1 and increased secretion of VCAM-1 and E-selectin	Predictors of prognosis and mortality rate
	NF-κB pathway, FGF23 pathway, NO/cGMP signal transduction pathway, ATP ion channel	Reduce myocardial reperfusion injury
	Excite C1 neurons/Increases sympathetic nerve activity and arterial blood pressure	Risk factors for atherosclerosis
Respiratory disease	Energy metabolism	Biomarker of severity of disease
	Inhibite IL-33/TGF-β, JNK, ERK, NF-κB	Predictors of the prognosis and mortality rate
	MRGPRX2-mediated inflammation	Indicators of diagnosis and therapeutic effect
Chronic liver disease	Energy metabolism	Predictors of prognosis and mortality rate
Kidney disease	Energy metabolism	Predictors of kidney injury and mortality rate
	PD-1/PD-L1 pathway, Sirtuin 3/AMPK-regulated autophagy	Indicators of therapeutic effect
Sepsis	Energy metabolism	Biomarker of severity of disease
	HMGB1 lactylation	Predictors of prognosis and mortality rate
	Inhibit NF-κB pathway-mediated production of inflammatory cytokines	
Arthritis	Energy metabolism	Indicators of diagnosis
	Slc5a12-inhibit binding of CXCR3 and CXCL10	
	Slc5a12/PKM2/STAT3/IL-17	

### Wound healing

Wound healing is a dynamic, complex biological process. Generally, wound healing can be divided into four overlapping stages, namely, hemostasis, inflammation, proliferation, and remodeling, which are regulated by various cytokines and growth factors^187^. Importantly, lactate is metabolized in mass during wound healing and plays an indispensable role because of the high levels of secreted cytokines and growth factors and the neovascularization that occurs upon immune system activation, resulting in increased metabolism and possibly hypoxia^188^. Thus, lactate, an energy substrate, can meet the high metabolic demands of wound healing. In addition, accumulated lactate reduces the pH of the alkaline environment caused by the reduction in carbon dioxide levels and the high oxygen tension, enabling cells to proliferate and differentiate within the optimized physiological pH range^189^. Hence, Trabold et al. suggested that lactate can be used as a substitute for oxygen to initiate healing^190^. Lactate also plays a role in promoting angiogenesis, as supported by the increased vascular endothelial growth factor (VEGF) levels and increased angiogenesis at oxygenated sites after the subcutaneous implantation of a matrix glue containing a lactate-releasing agent in mice^191^. In addition, some evidence indicates that lactate can stimulate fibroblasts to synthesize collagen in the ECM^192,193^. Lactate has been shown to stimulate vasogenic stem cells through the redox system^194^. Liu et al. demonstrated that lactate facilitated the activation of the transcription factor HIF-1α, which can regulate hypoxia^195^. Previously, Vural et al. found that HIF stimulated VEGF, promoted angiogenesis, and accelerated wound healing^196^. M2-polarized macrophages mediate wound healing through Akt, ERK1/2, and STAT3 pathway^197–200^, and the promoting effect of lactate on the polarization and function of M2 macrophages has been discussed.

It has been shown that lactate can promote wound healing. Preclinical trials have found that the improvement of wound healing in recombinant *L.reuteri* depends not only on the transformation of CXCL12 in the bacteria, but also on the combined presence of lactate produced by *L.Reuteri*^201,202^. Lactate production by *L.reuteri* alters the local wound environment, lowering pH, inhibiting the enzyme CD 26 that degrades CXCL12, and allowing increased bioavailability of CXCL12^203^.

### Ischemic injury

Tissue ischemia disrupts oxygen and glucose delivery, resulting in metabolic disorders^204^. Hypoxia directly leads to lactate accumulation, which lowers pH to activate ion transporters^205^, primarily Na^+^/H^+^ exchange (NHE) proteins that regulate intracellular pH by exchanging protons for extracellular sodium ions^206^. In acute tissue ischemia, NHE1 elevates intracellular Na^+^ levels, leading to increased Ca^2+^–Na^+^ exchange and intracellular calcium overload. During ischemia/reperfusion injury, lactate induced the release of TNF-α, IL-6, and IL-1 in the myocardium and brain, further exacerbating neuronal damage in acute stroke^207^. Inhibition of the lactate-activated GPR81 signaling pathway plays a protective role in ischemic brain injury^208^. In addition, evidence suggests that lactate treatment alleviated brain damage and improved behavior in rat models of hypoxic ischemia (HI)^209,210^, indicating that lactate may play a neuroprotective role in ischemic hypoxic encephalopathy. In a study of acute heart failure (AHF) in zebrafish, lactate acquired as an active component of herbal extracts was found to inhibit AHF, inflammation, and cardiac hypertrophy^211^. Moreover, increasing lactate levels during ischemia restored the M2-like polarization of macrophages in an MCT1-dependent manner, and an increase in VEGF production by polarized macrophages generated a positive feedback loop that further stimulated angiogenesis and ultimately facilitated postischemia revascularization and the regeneration of damaged muscle^132^.

As a product of tissue hypoxia, lactate itself can be used as an indicator of tissue hypoperfusion. Clinically, the determination of serum lactate plays a very important role in guiding the treatment of patients with ischemic injury, especially in the treatment of tissue hypoxia and ischemia-reperfusion injury caused by cerebral ischemia and insufficient blood flow in patients with myocardial infarction^212–215^. In addition, lactate is considered to be a predictor of major complications after cardiac surgery^216,217^, the significance of blood lactate measurement in monitoring the progress of cardiac surgery has been clarified.

### Tumor growth and metastasis

#### GPR81 and acidification function

Lactate and lactate-mediated activation of the GPR81 signaling pathway contribute to several facets of tumor progression, including cell proliferation, invasion, angiogenesis, immune tolerance, and immune cell escape from surveillance^6^. Because of their low perfusion, tumors are usually considered to reside in an isolated metabolic microenvironment, where local nutrient exchange predominates over circulating nutrient exchange. When the nutrient demand required for rapid tumor growth exceeds the energy supply, tumor growth depends on glycolysis, which produces a large amount of lactate. This process is called the Warburg effect, and lactate is considered a metabolic substrate that enables cancer cells to proliferate. Overexpression of TAp73, a key regulator of glycolysis, has been identified as a protector of tumor proliferation by promoting lactate production^218^. A large number of studies have recognized that lactate is a possible mediator of the loss of p53 signaling, which promotes tumor cell proliferation^219^. Lactate signals through the cell-surface receptor GPR81 in a process that is independent of MCT, protons, and cellular glucose metabolism but that plays an important role in tumor growth. Studies have shown that GPR81 expression increases in tumor cells in response to autocrine signaling by lactate; that is, the lactic acid produced by tumor cells activates GPR81 on tumor cells and produces an oncogenic phenotype^220^. In addition, lactate can act in a paracrine manner by activating GPR81 on nontumor cells in the TME to promote tumor growth, and tumor cell-derived lactate can activate GPR81 on dendritic cells and thus prevent tumor-specific antigen presentation to other immune cells^221^. A more specific study showed that GPR81 activation reduced the intracellular cAMP concentration, thereby decreasing the phosphorylation of the transcriptional activator TAZ in the Hippo pathway, promoting TAZ to enter the nucleus and bind TEAD1 (forming the TaZ-Tead1 complex), leading to the activation of PD-L1 expression to provide an effective means for tumor cells to escape the immune system^222^. In conclusion, lactate secreted by tumor cells acts on GPR81 of tumor cells or nontumor cells in the TME, ultimately affecting tumor cell function as well as information exchange and interactions between cells, all of which has the potential to affect tumor cell growth and proliferation. The flow of lactate out of tumor cells prevents further acidification of the intracellular environment but acidifies the extracellular environment. The mechanisms by which tumor cells can release H^+^ include the upregulation of NHE1 (Na^+^/H^+^ exchanger) and CAR9 (carbonic anhydrase IX)^223,224^. NHE1 promotes H^+^ efflux across the plasma membrane of cancer cells via a naturally occurring, internally oriented Na^+^ gradient. CAR9 catalyzes the conversion of extracellular water and carbon dioxide to carbonic acid, which is decomposed into bicarbonate and H^+^; then, sodium bicarbonate is transported into tumor cells through the sodium bicarbonate cotransporter HVCn1, leaving H^+^ outside. In addition, vacuolar (V)-type proton pumps in the tumor cell plasma membrane^225^ actively pump H^+^ out of the cell in a manner driven by direct binding and hydrolysis of ATP. Studies have confirmed that NHE1, HVCn1, and V-type proton pumps can promote the proliferation, migration, and drug resistance of tumor cells^225–234^, and silencing NHE1 or SLC4A7 significantly reduced tumor growth in a mouse xenograft model^235^. In conclusion, NHE1, CAR9, HVCn1, and V-type proton pumps act together on the plasma membrane of tumor cells to form a transmembrane pH gradient based on the inward H^+^ gradient. This pH regulation is crucial for tumor cell survival and proliferation. Therefore, the data support further investigation of these transporters, which promote cancer cell growth by exporting H^+^, as potential drug targets.

#### Tumor angiogenesis

We have previously discussed the role of lactate in promoting angiogenesis during wound healing. To a certain extent, the TME is similar to a trauma-related microenvironment. Lactate in the TME stimulates endothelial cell activation and angiogenesis through HIF-independent and HIF-dependent pathways. In the HIF-independent pathway, lactate is transported into cells via MCT1, wherein it is oxidized to pyruvate to produce NADH, which activates ROS production; as previously established, ROS production stimulates angiogenesis^236^. Another HIF-independent mechanism that facilitates angiogenesis involves the direct binding of lactate to NDRG3, a downstream regulatory protein in the N-Myc pathway; this binding event prevents NDRG3 degradation^237^. NDRG3 binds c-Raf to activate RAF-ERK signaling and promote angiogenesis under conditions of low oxygen tension and high lactate concentrations. HIF-1α has been reported to induce VEGF expression. The HIF-dependent pathway is based on the ability of lactate to stabilize HIF-1α under normoxic conditions. HIF-1α is a key regulator of the response to hypoxia^238,239^ that is continuously synthesized and degraded in the cytoplasm. Under normoxic conditions, proline hydroxylases (PHDs) are the main oxygen sensors in cells, and PHDs can hydroxylate HIF-1α at specific proline residues to promote the subsequent degradation of HIF-1α by the UPS. PHD, however, is inactivated under hypoxic conditions. This inactivation of PHD prevents the proteasomal degradation of HIF-1α, allowing HIF-1α to migrate to the nucleus, where it binds HIF-1β and promotes the transcription of many tumor-promoting genes^240^. Lactate has been reported to be absorbed by tumor cells and subsequently converted to pyruvate, which directly competes with α-KG to inhibit the activity of PHD, thereby stabilizing HIF-1α levels^240^. Hence, exogenous lactate stabilizes HIF-1α by acting as a substrate for pyruvate production, and a high lactate concentration in the TME ensures the transcriptional activation of tumor-promoting genes in all tumor cells regardless of oxygen supply.

#### Tumor invasion

Considerable evidence suggests that basal membrane (BM) remodeling^241^ and epithelial–mesenchymal transition (EMT)^242^ are two features of invasive tumors. Cancer-associated fibroblasts (CAFs) synthesize type I collagen, an important promoter of BM remodeling^243^. There is evidence that lactate promotes the proliferation and migration of CAFs and enhances the synthesis of type I collagen in CAFs^244^. In CAFs, COX-2 regulates the biosynthesis of type I collagen, and lactate was shown to increase COX-2 expression in a p38 kinase-dependent manner^245^. MMPs degrade collagen and glycoproteins and are the key collagenases that promote tumor invasion^246^. Lactate has been reported to promote LPS-stimulated MMP expression through the PLD/MAPK/NF-κB pathway^247^ and to increase caveolin1-induced MMP expression through the ERK/p90RSK pathway^248^. In conclusion, this evidence indicates that lactate may be a signaling molecule that increases MMP expression, leading to collagen degradation and the increased invasion of tumor cells. EMT is mainly activated by a cascade of specific factors/cytokines and related signaling pathways, and lactate has been shown to be involved in the TGF-β/Smad and Wnt/β-catenin signaling pathways, among others, that can activate EMT^249^.

#### Tumor immunity

Immune cells are an important component of the TME, and lactate is generally considered an immunosuppressive molecule that promotes malignant tumor growth due to the dependence on both H^+^ and lactate (Table 3). The pH in tumors can be as low as 5.6 but primarily ranges from 6.0 to 7.0^250^. The accumulation of lactate in the TME entails acidification of the TME, and a lower extracellular pH has been shown to impair almost all aspects of CD8^+^ and CD4^+^ T lymphocyte function, including the activation of cytotoxicity, chemotactic motility, and proliferation^251^. Experiments have shown that^252^ the accumulation of lactate in the TME entails acidification of the TME, and a lower extracellular pH has been shown to impair almost all aspects of CD8^+^ and CD4^+^ T lymphocyte function, including the activation of cytotoxicity, chemotactic motility, and proliferation^253,254^. During immune activation, the coactivator CD28 binds to the B7 receptor on antigen-presenting cells, stimulates the PI3K/Akt signaling pathway, and allows T cells to accelerate the synthesis of cytokines required for the immune response^255,256^. Specifically, for T cells, the activation of immune function directly coincides with metabolic reprogramming^257^; activated T cells and tumor cells both depend on glycolytic metabolism, and both must excrete lactate to avoid intracellular acidification. Due to the change in the lactate concentration gradient across the cell membrane, the accumulation of extracellular lactate from tumors prevents activated T cells from releasing lactate. In essence, high extracellular lactate in the TME leads to the accumulation of endogenous lactate within T cells, which reduces the secretion of proinflammatory cytokines^258^. However, this phenomenon is unique to activated cytotoxic T cells because in Tregs, the Treg-specific transcription factor FoxP3 inhibits c-Myc signaling to enable a switch to OXPHOS, thus keeping these cells active in the TME^158^. Alcinotto et al. used the proton pump inhibitor esomeprazole to restore normal pH and reverse the acid-induced impairment of tumor-specific CD8^+^ T lymphocytes in humans and mice^259^. Further studies confirmed that inhibition of the acidifier ion channel Ae2 or activation of the alkalization channel HVCn1 significantly improved T-cell function and the antitumor activity of Glypican 3 (GPC3)-specific CAR T cells in vitro^260^. In conclusion, the inhibition of lactate is a potential strategy for overcoming T-cell dysfunction under acidic pH conditions that could enhance adoptive T-cell transfer-based immunotherapy. Lactate itself has been found to mediate mechanisms of immunosuppression. High lactate production by tumor cells increases the acidity of the TME. The hypoxic TME induces the HIF-1α-mediated upregulation of PD-L1, leading to CD8^+^ T-cell dysfunction^261^. Studies have also found that SLC43A2, a methionine transporter in tumor cells, disrupts methionine metabolism in CD8^+^ T cells and reduces the methylation of T-cell histones, thereby inhibiting T-cell-mediated immunity^262^. Lactate was found to inhibit nuclear factor of activated T cells (NFAT), leading to the inactivation of tumor-infiltrating CD8^+^ T cells and NK cells and reduced IFNγ production^263^. Therefore, some researchers have speculated that lactate could ultimately promote the differentiation of Th1 and Th2 effector cells through signal transduction and metabolic regulation while inhibiting Treg induction and T-cell inactivation^264,265^. It is worth mentioning that a recent study found that in tumors, the metabolite lactate affected the TME by regulating the lactylation of MOESIN in Tregs and enhancing TGF-β signal transduction, thus promoting a new mechanism of tumorigenesis^266^. Regarding innate immunity, lactate can increase the expression of VEGF and ARG1 in TAMs, polarizing them toward the immunosuppressive and protumorigenic M2 phenotype^156^. The ability of lactate to mediate this process has been thought to depend on MCTs and HIF-1α stability. However, the subsequent discovery of histone lactate modification suggests a new mechanism, namely, histones can be modified by lactatelto regulate the polarization state of TAMs through increased expression of ARG1 and other TAM-related genes; thus, TAMs function through multiple mechanisms to disrupt antitumor immune responses, and their presence is considered a negative prognostic marker^267^. TAMs produce IL-10, an immunosuppressive cytokine^268^. TAMs also express the checkpoint inhibitor PD-L1, which binds to its receptor PD-1 on activated T lymphocytes and drives cell apoptosis^269^. In addition, TAMs express CCL22, attract Tregs to increase the production of molecules such as PD-L1, disrupt T-cell metabolism by expressing ARG1, and deprive T cells of L-arginine, a key nutrient for T-cell growth^270^. Therefore, lactate-driven TAM polarization is a key mechanism of malignant tumor cell immune escape. Lactate and lactylation are also associated with the release of high mobility group box protein 1 (HMGB1), a damage-associated molecular pattern (DAMP), from activated macrophages^91,271^. Emerging evidence suggests that DAMPs, including HMGB1, play an important role in initiating and sustaining chronic inflammation that impairs antitumor immunity and promotes cancer progression^272,273^. However, HMGB1 is also thought to have antitumor properties, and interestingly, it inhibits glycolysis in tumor cells, leading to metabolic cell death^274^. Therefore, it is necessary to clarify the effects of DAMPs such as HMGB1 on tumor metabolism and their dual roles in promoting or inhibiting cancer progression.

**Table 3:** Effects of lactate on immune cells in tumor microenvironment

Cell type	Mechanism	Effect
macrophages	Activation of the ERK-STAT3 pathway	M2 polarization (↑IL-6, ↑VEGF, ↑ARG1, ↑CCL5)
	Activation of GPR132 and Notch	
	HIF1α stablization	
	Histone lactylation	
T cell	Acidic pH environment	↓Effector function
	Activation PD-L1/PD-1 pathway	↓Proliferation
	Inhibition of p38 and JNK–JUN	↑PD-1
	Inhibition of lactate efflux	↑Apoptosis
	Reduced NAD availability	↓Cytokine production
Dendritic cell	Acidic pH environment	↓Differentiation
	Reduced CD1a and increased CD14 expression	↓IL-12
	Activation of GPR81 and import via SLC16A	↓IL-6
		↓TNF
		↑Kynurenine
Treg cell	FOXP3-mediated repression of MYC and modulation of LDH	↑Proliferation
	Sustained fatty acid synthesis through ACC	↑Differentiation
		↑TGFβ
		↑IL-10
NK cell	Inhibition of NFAT and NKp46	↓Cytolytic function
	Inhibition of mTOR signaling	↓IFNγ
	Acidic pH environment	↑Apoptosis
	HDAC inhibition	

#### Clinical significance

Previous studies have highlighted how lactate creates a conducive microenvironment for tumor cell growth and represents an energy course for tumor cells. Lactate promotes tumor growth in a number of ways. On the one hand, lactate inhibits immune cell effects in the TME. Lactate blocks the activity of MCT1, a lactate transporter in immune cells in the TME^275^, to prevent the removal of lactate from cells and inhibits the proliferation and survival of immune cells^263^. Moreover, lactate-mediated signaling activates tumor cell proliferation and drug resistance through GPRs that enhance the expression of PD-L1^222^. Lactate also prevents dendritic cells in the TME from presenting tumor-specific antigens to immune cells^221^. On the other hand, lactate can be directly absorbed and metabolized by tumor cells to promote the TCA cycle^41,42^, and as a signaling factor that regulates the hypoxia response, lactic acid promotes tumor cell growth^237^. In view of its immunosuppressive role in promoting angiogenic tumor invasion and metastasis, lactate is considered an indicator of high malignancy and poor prognosis in several types of cancer. In lung cancer, elevated systemic lactate is a negative prognostic factor for metastatic NSCLC and is associated with significantly shorter overall survival^276^. Wei et al. found that serum lactate levels were higher in patients with metastatic colorectal cancer than in patients without systemic disease^277^. In addition, studies have reported that increased lactate levels are negatively correlated with the percentages of Th1 cells and cytotoxic T lymphocytes (CTLs) in tumors, reflecting the impaired immune capacity in the TME. In head and neck cancer, an increased tumor lactate concentration was identified as a predictor of subsequent lymph node metastasis or distant metastasis^278^. New techniques, such as nuclear magnetic resonance spectroscopy (MRS) and hyperpolarized (HP) ^13^C-MRI, are changing the prospects of measuring intracellular lactate concentration and increasing the feasibility of using this concentration as a tumor biomarker^279–282^. MRS has been used to show that an elevated lactate level within tumors is a poor prognostic indicator in breast cancer^283^. In addition, in HER2-positive breast cancer, elevated intratumoral lactate levels are associated with HER2 addiction status and susceptibility to trastuzumab, a HER2 inhibitor. Therefore, lactate can be used as a predictive biomarker for the optimal prescription of HER2-targeted drugs in this patient population^284^. MRS-based detection of lactate predicted the poor prognosis of patients with diffuse endogenous pontine glioma^285^. Therefore, the future development of metabolic imaging must consider the different metabolic phenotypes of tumors. Current studies on the application of lactic acid imaging as a biomarker for tumor diagnosis include NCT01881386 (the use of MRS to observe changes in tumor lactic acid levels in vivo in response to treatment), NCT04584827 (the effects of lactic acid levels on mortality and morbidity in patients undergoing intracranial mass surgery under general anesthesia), NCT03129776 (HP ^13^C-MRI of lactic acid in cervical tumors to identify areas of radiation resistance and to guide radiotherapy), and NCT03531307 (the association of lactic acid levels with the tumor proliferation marker Ki67 in patients with brain tumors).

#### Therapeutic targets

Studies have confirmed that glycolysis accelerates the metabolic switch associated with increased lactate production in tumor cells as a result of the increased expression of oncogenes, mainly c-Myc and HIF-1α^286^. The conversion of intracellular pyruvate to lactate is catalyzed by LDH, a heteromeric protein composed of two subunits each of LDH-A and LDH-B that exists in five isomeric forms. LDH-5 (A4) has a higher affinity for pyruvate than for lactate, which is conducive for the conversion of pyruvate to lactate, while LDH-1 (B4) has a higher affinity for lactate than for pyruvate, which is conducive for the conversion of lactate to pyruvate. In general, the induction of LDH-A and silencing of LDH-B leads to increased LDH-5 activity, decreased LDH-1 activity, and an increase in intracellular lactic acid production. The malignant proliferative and invasive potential of tumors has been shown to be dependent on LDH-A activity^287^, and the activation of HIF-1α and c-Myc and the inhibition of p53 regulate LDH-A gene transcription^288^. LDH-B is inhibited by epigenetic mechanisms such as DNA methylation^289–292^. The priority of glycolysis is to convert pyruvate into lactate. Because LDH-A is increased in cancer and is responsible for generating the main form of lactate, LDH-A is a potential drug target for cancer treatment. In fact, a large number of studies have shown that the inhibition of LDH-A both in vivo and In vitro can block tumor growth and invasion^293–297^. FX-11, a selective LDH-A inhibitor, has shown antitumor activity in xenograft tumor models^298,299^ and has potential for development as a cancer treatment. Galloflavin inhibited LDH-A activity^300^ and thus inhibited lactate production and proliferation in Burkitt lymphoma cells^301^. Chronic stress-induced epinephrine alters the pH to promote cancer stem cell-like properties in breast cancer cells through lactic acid production involving LDH-A; as vitamin C can inhibit LDH-A, it may be an effective therapeutic in stress-related breast cancer^302^. LDH-A is considered a safe target for cancer treatment because the loss of LDH-A protein due to an inherited deletion in the LDHA gene results in only mild myopathy^303,304^. Theoretically, blocking lactate production in tumor cells by inhibiting LDH-A could have some unmanageable adverse effects. For example, the intracellular accumulation of pyruvate and NADH drives ECM remodeling by inducing collagen hydroxylation, which results in increased collagen stability and thereby promotes the metastatic growth of breast cancer cells^305^. As pyruvate is catalyzed to lactate, a molecule of NADH is oxidized to NAD^+^; blocking these reactions directly leads to a lack of NAD^+^ for glycolysis, which slows the reaction rate. In addition, the production and activity of lactic acid play significant roles in the maintenance of cellular and biological functions and immune regulation. Therefore, even though the anticancer potential of LDH-A has been confirmed, LDH-A blockade may have many nontarget effects; these potential challenges must be overcome in the application of LDH-A inhibitors for the treatment of cancer.

The regulation of lactate by c-Myc and HIF-1α in tumors can also be achieved by the induction of MCT; c-Myc induces MCT1^306,307^, and HIF-1α induces MCT1 and MCT4^308^, the major lactate transporters utilized by cancer cells. Blocking MCT1 and MCT4 function or reducing their density in the plasma membrane are potential strategies for cancer treatment^309^. The rationale is that if the outlet for lactic acid in cancer cells is blocked or reduced, the resulting intracellular acidification will kill the cell; importantly, selective inhibitors of MCT1/4 have been shown to be effective cancer treatments in preclinical studies^310^. Syrosingopine is an effective dual inhibitor of MCT1 and MCT4^311^ that has been proven to sensitize cancer cells to metformin in vivo^312^, thus enhancing its antitumor activity; these data suggest the potential for syrosingopine as an adjunctive therapy for future clinical anticancer drugs. AZD3965, a dual inhibitor of MCT1 and MCT2, was shown in both in vitro and in vivo preclinical studies to be safe for breast cancer treatment^313^. A phase I clinical trial of AZD3965 in patients with advanced solid tumors and B-cell lymphoma has been completed (NCT01791595). MCT1 and MCT4 transport to the cell surface after expression depends on the glycoprotein chaperone CD147^314^. Therefore, CD147 has become a potential drug target to disrupt MCT membrane insertion and function in cancer therapy.

There are several possible antitumor therapies that aim to inhibit lactate production (Table 4): 2-deoxyglucose (2-DG), which can compete with glucose in vivo, reduces lactate production in tumor cells, and inhibits rapid tumor cell growth by reducing glucose utilization^315^. Phase I clinical trials have been conducted to evaluate the dosage of 2-DG in patients with several types of solid tumors, including prostate cancer and glioblastoma, based on preclinical efficacy^316^. Breast cancer cells were treated with 2-DG as a metformin adjuvant, and the results indicated a synergistic effect on growth arrest^317^. In addition, 2-DG enhanced CD8^+^ memory cell formation and antitumor function, including increasing the homing of lymphocytes to lymph nodes, IFN-γ and TNF-α production, and tumor regression in mice with melanoma^318^. These results suggest that glucometabolic modulators can exert certain antitumor effects alone or as adjuvants to traditional drugs. Lonidamine, a dechlorinated derivative of indolizazol-3-carboxylic acid, strongly inhibits oxygen consumption, aerobic glycolysis, and lactate transport and accumulation in tumor cells. Because its specific mechanism of action and side effect profiles do not overlap with those of standard antineoplastic drugs, lonidamine combined with standard chemotherapy for the treatment of solid tumors has been widely studied. Lonidamine was confirmed to increase the toxicity of anthracycline-based drugs in human breast cancer cell lines and to enhance cisplatin activity in platinum-sensitive and platinum-resistant ovarian cancer cell lines. The results from phase II-III clinical trials of lonidamine for advanced breast, ovarian and lung cancer are encouraging. Dichloroacetate (DCA) inhibits pyruvate dehydrogenase kinase, increases glucose uptake into mitochondrial oxidation, and decreases lactate production. The results of a phase I clinical trial showed that long-term oral DCA treatment was feasible and well tolerated in patients with relapsed glioblastoma and other tumors that have metastasized to the brain^319^. The results from another phase II study of DCA in combination with chemoradiotherapy in unresected locally advanced squamous cell carcinoma of the head and neck showed that the addition of DCA to cisplatin-based chemoradiation (CRT) appeared to be safe and had no adverse effect on survival or expected metabolite changes. These data provide support for further studies on combinations of metabolic drugs with CRT^320^.

**Table 4:** Drugs that target production and transport of lactate

Molecule	Target	Mechanism	Condition	Clinical trial
AZD3965	Lactate transporters	Inhibits MCT1/2	Malignant tumor	Phase I Trial
α-Cyano-4-hydroxycinnamate	Lactate transporters	Inhibits MCT1/2	Hyperglycolytic malignancies	N/A
AR-C155858	Lactate transporters	Inhibits MCT1/2	Hyperglycolytic malignancies	N/A
Syrosingopine	Lactate transporters	Inhibits MCT1/4	Malignant tumor	N/A
Meplazumab	CD147	Inhibits the distribution of MCT1/4 on cell membrane	Malignant tumor, COVID-19	Phase I Trial
Stiripentol	LDH	Inhibits LDH	Epilepsy, Dravet Syndrome	Approved by FDA
Galloflavin	LDH	Binds the free enzyme	Malignant tumor	N/A
N-hydroxyindoles	LDH	Compete with pyruvate and NADH	N/A	N/A
AT-101 (gossypol)	LDH	Inhibits LDH	Malignant tumor	Phase II Trial
FX-11	LDH	Inhibits LDH-A	Pancreatic cancer	N/A
GSK2837808A	LDH	Inhibits LDH-A	N/A	N/A
Vitamin C	LDH	Inhibits LDH-A	Breast cancer	N/A
2-DG	HK	Inhibits glycolysis by competing with glucose	Malignant tumor	Dose Escalation Trial, Phase I/II Trial
DCA	PDK	Increases glucose uptake into mitochondria	Malignant tumor, Lactic acidosis	Phase I/II Trial
Lonidamine	HK	Inhibits glycolysis	Malignant tumor	Phase II Trial

Notes: *N/A* not available

## Conclusions and perspectives

Two ATP production systems are functional in mammalian cells: glycolysis and OXPHOS. Glycolysis does not require oxygen, and the final product of this pathway is lactate. Less ATP is generated through glycolysis than through OXPHOS. Therefore, in textbooks, glycolysis has been described as a supplementary method of ATP production under anaerobic conditions, and historically, lactate has been considered a waste product that helps maintain temperature and generate heat produced by muscle movement; hence, lactate is known to cause muscle fatigue. However, a “lactate revolution” in the 1970s led researchers to realize that lactate, at the intersection of anaerobic and aerobic carbohydrate metabolism, is a direct and critical component in the cellular energy repository and that the steps through which lactate leads to ATP production are simpler and faster than those involved in the oxidative utilization of glucose. Subsequent studies gradually revealed that lactate is a major renewable carbohydrate fuel in mammals and a recyclable redox buffer that maintains a balanced cellular redox state in tissues throughout the body. Thus, the roles of lactate as a metabolic feedback regulator and unique signaling molecule were considered, and the function of lactylation as a novel PTM of proteins has been explored. Therefore, lactate is recognized to play a significant role in many physiological and pathological processes, including the regulation of energy metabolism, immunity responses, memory formation, wound healing, and tumor development. These processes are regulated by lactate as a signaling molecule or through lactylation, in addition to its role as a metabolic substrate. Currently, lactate is mostly used as an indicator for disease diagnosis, prognosis, and efficacy evaluation in the clinical (Table 2). Lactate has rarely been used as a therapeutic target in clinical practice, and studies focus more on the therapeutic potential of small nucleic acid drugs that target cell function^321–323^. As a plasma biomarker of disease, lactate theoretically has potential to be a molecule that stimulates specific-responsive drugs as same as glucose^324^. With the exploration of its functions in signal transduction and post-translational modification of proteins, lactate is expected to become a valuable target for the treatment of chronic cardiovascular diseases, nerve injury, inflammatory disease, and tumors.

As a necessary product of glycolysis, lactate has continued to be the subject of study for reasons that include abnormally elevated levels in inflammatory diseases and the TME. Since the discovery of the Warburg effect, glycolysis and lactate production are no longer considered specific to hypoxia, which better explains disease heterogeneity. This recognition has also greatly improved the understanding of inflammatory diseases and cancer. Advances in research have revealed the metabolic regulation of cell function, the adaptability of cells to different environments, and the feedback regulation of metabolism by metabolites. The breakthrough in discovering the epigenetic function of lactate as a substrate of histone Kla seems to represent a fulcrum of metabolism and gene regulation.

PTMs are important epigenetic mechanisms that regulate cell function. With the rapid development of high-resolution MS technology in recent years, various novel acylation pathways have been identified in succession, including formylation, propionylation, butyrylation, crotonylation, 2-hydroxyisobutyrylation, β-hydroxybutyrylation (bhb), succinylation, malonylation, glutarylation, benzoylation, and lactylation. Among the known classical HATs, p300/CBP has been identified as a mediator of the most diverse acyltransferase activities^325^. Given the functional specificity of acyl modifications, what are the main factors that determine the specificity and selectivity of p300/CBP activity? In other words, how does a nonspecific HAT specifically catalyze lactylation? First, dynamic changes in intracellular acyl-CoA concentrations are clearly involved^100^. However, since the concentration of acetyl-CoA is higher than that of other acyl-CoAs in most cases, more studies are needed to clarify the regulatory mechanisms. Second, acyltransferase activity differs by organelle, and this preference is an important factor. For example, recently identified Kbhb substrates are more highly concentrated in the nucleus; therefore, acyltransferases in the nucleus seem to interact preferentially with Kbhb^326^. Another interesting question that has yet to be answered is: what is the relationships among simultaneous different PTMs? Acylation is realized through cellular metabolites that are acyl donors, such as acetyl-CoA, succinyl-CoA and lactyl-CoA, and histone acylation can usually activate transcription and regulate gene expression^327^. Previous studies have shown that acetylation and succinylation show a preference for lysine residues at different locations in the same protein. However, two or more different PTMs can often be observed at some of the same lysine residues, and different modifications have also been found at certain sites of proteins in the central metabolic pathway^328^. For example, in Glis1-induced histone modification, p300 has been shown to increase both the acetylation of H3K27 and the lactylation of H3K18 in a second wave of PTMs at pluripotent gene promoters, which activates gene expression and facilitates somatic reprogramming^98^. Moreover, studies of Kla in rice have found other types of acylation simultaneously with lactylation on the same lysine residues of the same proteins, suggesting the potential for crosstalk between these modifications^329^. These results indicate coordinated interactions between different PTMs in cells. In fact, it has been proposed that two different types of PTMs target different lysine residues or that PTM machineries compete to modify the same lysine. However, the mechanism through which different acyl modifications coordinate and coregulate cell physiology and biochemistry has not been clarified in vivo or in vitro. In some cases, PTMs can be established in a nonenzymatic manner^330^. Notably, the enzymatic and nonenzymatic deposition of the same PTM is coordinated. Organelles may play a decisive role in the way in which different modifications are rendered, and nonenzymatic regulation has been proposed as the main mechanism of mitochondrial acetylation^331^. Interestingly, the regulation of cell function through the same modification may involve a division of labor between enzymatic and nonenzymatic regulators. Nonenzymatic lactylation is enriched through the glycolysis pathway, which can inhibit enzymatic activity and reduce glycolysis metabolites^102^, while enzyme-regulated lactylation is enriched in inflammatory pathways and can regulate inflammatory homeostasis^16^. Unfortunately, it remains unknown whether this division of labor is an accurate description or whether the limited number of related studies has led to an inaccurate understanding. In general, the complexity of PTMs is far beyond our current understanding, and researchers’ knowledge of PTMs is currently limited. However, recent studies have provided insight into some aspects of PTMs and suggested worthwhile future directions of exploration in the field of life science.

Kla in tumor tissues generally promotes tumor immune evasion by inducing the anti-inflammatory phenotype of TAMs. Moreover, it may directly regulate oncogene expression to promote tumor growth, metastasis, and invasion. Lactylation also regulates gene expression in macrophages, maintains immune homeostasis, and plays a vital role in inflammation, repair, and wound healing. Therefore, lactylation holds great potential as a therapeutic target in inflammation, cancer, and systemic diseases caused by immunosuppression. Furthermore, lactylation also plays a potential role in regulating neuronal excitation. However, the specific underlying mechanism leading to such neuronal excitation remains unexplored. The function and specific mechanisms of lactylation and its regulatory enzymes in exercise, lipolysis, neuroprotection, angiogenesis, and other aspects also need to be investigated, and these data will provide new diagnostic and treatment strategies for many chronic diseases, such as atherosclerosis and AD. Specifically, studies have revealed the existence and function of nonhistone Kla in plants^329,332^, and Kla has considerable potential as a target for development in agriculture.

## References

[1] Ferguson BS (2018). Lactate metabolism: Historical context, prior misinterpretations, and current understanding. Eur. J. Appl Physiol..

[2] Brooks GA (2002). Lactate shuttles in nature. Biochem Soc. Trans..

[3] Brooks GA (2009). Cell-cell and intracellular lactate shuttles. J. Physiol..

[4] Daw CC (2020). Lactate elicits ER-mitochondrial Mg(2+) dynamics to integrate cellular metabolism. Cell.

[5] Zhao Y (2020). HCAR1/MCT1 regulates tumor ferroptosis through the lactate-mediated AMPK-SCD1 activity and its therapeutic implications. Cell Rep..

[6] Brown TP, Ganapathy V (2020). Lactate/GPR81 signaling and proton motive force in cancer: Role in angiogenesis, immune escape, nutrition, and Warburg phenomenon. Pharm. Ther..

[7] Felmlee MA (2020). Monocarboxylate transporters (SLC16): Function, regulation, and role in health and disease. Pharm. Rev..

[8] Liberti MV, Locasale JW (2016). The Warburg effect: How does it benefit cancer cells?. Trends Biochem. Sci..

[9] Vaupel P, Multhoff G (2021). Revisiting the Warburg effect: Historical dogma versus current understanding. J. Physiol..

[10] Li X (2016). Mitochondria-translocated PGK1 functions as a protein kinase to coordinate glycolysis and the TCA cycle in tumorigenesis. Mol. Cell.

[11] Li M (2021). DDIT3 directs a dual mechanism to balance glycolysis and oxidative phosphorylation during glutamine deprivation. Adv. Sci..

[12] Teng R (2019). HSP60 silencing promotes Warburg-like phenotypes and switches the mitochondrial function from ATP production to biosynthesis in ccRCC cells. Redox Biol..

[13] Certo M (2021). Lactate modulation of immune responses in inflammatory versus tumour microenvironments. Nat. Rev. Immunol..

[14] Chen Z, Liu M, Li L, Chen L (2018). Involvement of the Warburg effect in non-tumor diseases processes. J. Cell Physiol..

[15] Dabral S (2019). A RASSF1A-HIF1alpha loop drives Warburg effect in cancer and pulmonary hypertension. Nat. Commun..

[16] Zhang D (2019). Metabolic regulation of gene expression by histone lactylation. Nature.

[17] Tong Y (2021). SUCLA2-coupled regulation of GLS succinylation and activity counteracts oxidative stress in tumor cells. Mol. Cell.

[18] Guo Y (2020). NF- kappa B/HDAC1/SREBP1c pathway mediates the inflammation signal in progression of hepatic steatosis. Acta Pharm. Sin. B.

[19] Lv L (2011). Acetylation targets the M2 isoform of pyruvate kinase for degradation through chaperone-mediated autophagy and promotes tumor growth. Mol. Cell.

[20] Galvan-Pena S (2019). Malonylation of GAPDH is an inflammatory signal in macrophages. Nat. Commun..

[21] Harmer AR (2008). Sprint training increases muscle oxidative metabolism during high-intensity exercise in patients with type 1 diabetes. Diabetes Care.

[22] Levy B (2005). Relation between muscle Na+K+ATPase activity and raised lactate concentrations in septic shock: a prospective study. Lancet.

[23] Fantin V, St-Pierre J, Leder P (2006). Attenuation of LDH-A expression uncovers a link between glycolysis, mitochondrial physiology, and tumor maintenance. Cancer Cell.

[24] Bennis Y (2020). A study of associations between plasma metformin concentration, lactic acidosis, and mortality in an emergency hospitalization context. Crit. Care Med..

[25] Jha M, Lee I, Suk K (2016). Metabolic reprogramming by the pyruvate dehydrogenase kinase-lactic acid axis: Linking metabolism and diverse neuropathophysiologies. Neurosci. Biobehav. Rev..

[26] Soreze Y (2013). Mutations in human lipoyltransferase gene LIPT1 cause a Leigh disease with secondary deficiency for pyruvate and alpha-ketoglutarate dehydrogenase. Orphanet J. Rare Dis..

[27] Luengo A (2021). Increased demand for NAD relative to ATP drives aerobic glycolysis. Mol. Cell.

[28] Emhoff C (2013). Gluconeogenesis and hepatic glycogenolysis during exercise at the lactate threshold. J. Appl. Physiol..

[29] DeBerardinis RJ (2007). Beyond aerobic glycolysis: Transformed cells can engage in glutamine metabolism that exceeds the requirement for protein and nucleotide synthesis. Proc. Natl Acad. Sci. USA.

[30] Halestrap AP (2013). The SLC16 gene family-structure, role, and regulation in health and disease. Mol. Asp. Med..

[31] Madaan A (2017). Lactate produced during labor modulates uterine inflammation via GPR81 (HCA). Am. J. Obstet. Gynecol..

[32] Sun Z (2019). Activation of GPR81 by lactate inhibits oscillatory shear stress-induced endothelial inflammation by activating the expression of KLF2. IUBMB Life.

[33] Wu G (2022). The lactate receptor GPR81 mediates hepatic lipid metabolism and the therapeutic effect of metformin on experimental NAFLDs. Eur. J. Pharmacol..

[34] Laroche, S. et al. Participation of L-lactate and its receptor HCAR1/GPR81 in neurovisual development. *Cells* **10**, 1640 (2021). 10.3390/cells10071640 PMC8303161 34208876

[35] Lu J, Tan M, Cai Q (2015). The Warburg effect in tumor progression: Mitochondrial oxidative metabolism as an anti-metastasis mechanism. Cancer Lett..

[36] Rabinowitz J, Enerbäck S (2020). Lactate: The ugly duckling of energy metabolism. Nat. Metab..

[37] Dienel GA (2019). Brain glucose metabolism: Integration of energetics with function. Physiol. Rev..

[38] Schurr A, West CA, Rigor BM (1988). Lactate-supported synaptic function in the rat hippocampal slice preparation. Science.

[39] Lhomme, T. et al. Tanycytic networks mediate energy balance by feeding lactate to glucose-insensitive POMC neurons. *J. Clin. Invest*. **131**, e140521 (2021). 10.1172/JCI140521 PMC8439611 34324439

[40] Gomez-Valades AG (2021). Mitochondrial cristae-remodeling protein OPA1 in POMC neurons couples Ca(2+) homeostasis with adipose tissue lipolysis. Cell Metab..

[41] Hui S (2017). Glucose feeds the TCA cycle via circulating lactate. Nature.

[42] Faubert B (2017). Lactate metabolism in human lung tumors. Cell.

[43] Titov DV (2016). Complementation of mitochondrial electron transport chain by manipulation of the NAD+/NADH ratio. Science.

[44] Ying W (2008). NAD+/NADH and NADP+/NADPH in cellular functions and cell death: regulation and biological consequences. Antioxid. Redox Signal.

[45] Tilton WM, Seaman C, Carriero D, Piomelli S (1991). Regulation of glycolysis in the erythrocyte: Role of the lactate/pyruvate and NAD/NADH ratios. J. Lab Clin. Med..

[46] Quinn WJ (2020). Lactate limits T cell proliferation via the NAD(H) redox state. Cell Rep..

[47] Wang C (2016). Malate-aspartate shuttle inhibitor aminooxyacetic acid leads to decreased intracellular ATP levels and altered cell cycle of C6 glioma cells by inhibiting glycolysis. Cancer Lett..

[48] Luengo A (2021). Increased demand for NAD(+) relative to ATP drives aerobic glycolysis. Mol. Cell.

[49] Patgiri A (2020). An engineered enzyme that targets circulating lactate to alleviate intracellular NADH:NAD(+) imbalance. Nat. Biotechnol..

[50] Perry JJ, Shin DS, Getzoff ED, Tainer JA (2010). The structural biochemistry of the superoxide dismutases. Biochim. Biophys. Acta.

[51] Shadel GS, Horvath TL (2015). Mitochondrial ROS signaling in organismal homeostasis. Cell.

[52] Corkey BE, Deeney JT (2020). The redox communication network as a regulator of metabolism. Front. Physiol..

[53] Yang S, Lian G (2020). ROS and diseases: Role in metabolism and energy supply. Mol. Cell Biochem..

[54] Jia L (2021). Rheb-regulated mitochondrial pyruvate metabolism of Schwann cells linked to axon stability. Dev. Cell.

[55] Benjamin D (2018). Dual inhibition of the lactate transporters MCT1 and MCT4 is synthetic lethal with metformin due to NAD+depletion in cancer cells. Cell Rep..

[56] Hoy AJ, Nagarajan SR, Butler LM (2021). Tumour fatty acid metabolism in the context of therapy resistance and obesity. Nat. Rev. Cancer.

[57] Pucino V, Bombardieri M, Pitzalis C, Mauro C (2017). Lactate at the crossroads of metabolism, inflammation, and autoimmunity. Eur. J. Immunol..

[58] Pucino V (2019). Lactate buildup at the site of chronic inflammation promotes disease by inducing CD4(+) T cell metabolic rewiring. Cell Metab..

[59] Liu L (2017). The Glia-neuron lactate shuttle and elevated ROS promote lipid synthesis in neurons and lipid droplet accumulation in glia via APOE/D. Cell Metab..

[60] Jin ES, Sherry AD, Malloy CR (2015). Lactate contributes to glyceroneogenesis and glyconeogenesis in skeletal muscle by reversal of pyruvate kinase. J. Biol. Chem..

[61] Lund J (2018). Utilization of lactic acid in human myotubes and interplay with glucose and fatty acid metabolism. Sci. Rep..

[62] San-Millan I, Brooks GA (2018). Assessment of metabolic flexibility by means of measuring blood lactate, fat, and carbohydrate oxidation responses to exercise in professional endurance athletes and less-fit individuals. Sports Med..

[63] de Boer, E. et al. Decreased fatty acid oxidation and altered lactate production during exercise in patients with post-acute COVID-19 syndrome. *Am. J. Respir. Crit. Care Med.* **205**, 126–129 (2022). 10.1164/rccm.202108-1903LE PMC8865580 34665688

[64] Fritzen AM, Lundsgaard AM, Kiens B (2020). Tuning fatty acid oxidation in skeletal muscle with dietary fat and exercise. Nat. Rev. Endocrinol..

[65] Wang, T. et al. Acetyl-CoA from inflammation-induced fatty acids oxidation promotes hepatic malate-aspartate shuttle activity and glycolysis. *Am. J. Physiol. Endocrinol. Metab.* **315**, E496–E510, (2018). 10.1152/ajpendo.00061.2018 29763372

[66] Brooks GA (2018). The science and translation of lactate shuttle theory. Cell Metab..

[67] Wasserman K (1985). Lactate, pyruvate, and lactate-to-pyruvate ratio during exercise and recovery. J. Appl Physiol..

[68] Brooks, G. J. C. M. The science and translation of lactate shuttle theory. *Cell Metab.* **27**, 757–785, (2018). 10.1016/j.cmet.2018.03.008 29617642

[69] Sahlin K, Fernstrom M, Svensson M, Tonkonogi M (2002). No evidence of an intracellular lactate shuttle in rat skeletal muscle. J. Physiol..

[70] Hashimoto T (2008). Evidence for the mitochondrial lactate oxidation complex in rat neurons: demonstration of an essential component of brain lactate shuttles. PLoS One.

[71] Hashimoto T, Brooks GA (2008). Mitochondrial lactate oxidation complex and an adaptive role for lactate production. Med. Sci. Sports Exerc..

[72] Hashimoto T, Hussien R, Brooks GA (2006). Colocalization of MCT1, CD147, and LDH in mitochondrial inner membrane of L6 muscle cells: evidence of a mitochondrial lactate oxidation complex. Am. J. Physiol. Endocrinol. Metab..

[73] Brooks GA (1991). Decreased reliance on lactate during exercise after acclimatization to 4300 m. J. Appl Physiol..

[74] Brooks GA, Gaesser GA (1980). End points of lactate and glucose metabolism after exhausting exercise. J. Appl. Physiol. Respir. Environ. Exerc Physiol..

[75] Brooks GA (1998). Mammalian fuel utilization during sustained exercise. Comp. Biochem. Physiol. B Biochem. Mol. Biol..

[76] Brooks GA (2021). Role of the heart in lactate shuttling. Front. Nutr..

[77] Gizak A, McCubrey JA, Rakus D (2020). Cell-to-cell lactate shuttle operates in heart and is important in age-related heart failure. Aging.

[78] Sun Y (2020). Modulation of the astrocyte-neuron lactate shuttle system contributes to neuroprotective action of fibroblast growth factor 21. Theranostics.

[79] Bisetto S (2019). New insights into the lactate shuttle: Role of MCT4 in the modulation of the exercise capacity. iScience.

[80] Sheikh-Hamad D (2021). Hints for a kidney lactate shuttle and lactomone. Am. J. Physiol. Ren. Physiol..

[81] Brooks GA (2020). The tortuous path of lactate shuttle discovery: From cinders and boards to the lab and ICU. J. Sport Health Sci..

[82] Irizarry-Caro RA (2020). TLR signaling adapter BCAP regulates inflammatory to reparatory macrophage transition by promoting histone lactylation. Proc. Natl Acad. Sci. USA.

[83] Troutman TD (2012). Role for B-cell adapter for PI3K (BCAP) as a signaling adapter linking Toll-like receptors (TLRs) to serine/threonine kinases PI3K/Akt. Proc. Natl Acad. Sci. USA.

[84] Matsumura T (2010). Identification of BCAP-(L) as a negative regulator of the TLR signaling-induced production of IL-6 and IL-10 in macrophages by tyrosine phosphoproteomics. Biochem. Biophys. Res. Commun..

[85] Sun S (2021). Lactic acid-producing probiotic saccharomyces cerevisiae attenuates ulcerative colitis via suppressing macrophage pyroptosis and modulating gut microbiota. Front. Immunol..

[86] Cui H (2021). Lung myofibroblasts promote macrophage profibrotic activity through lactate-induced histone lactylation. Am. J. Respir. Cell Mol. Biol..

[87] Pan RY (2022). Positive feedback regulation of microglial glucose metabolism by histone H4 lysine 12 lactylation in Alzheimer’s disease. Cell Metab..

[88] Yu J (2021). Histone lactylation drives oncogenesis by facilitating m(6)A reader protein YTHDF2 expression in ocular melanoma. Genome Biol..

[89] Smith EA, Hodges HC (2019). The spatial and genomic hierarchy of tumor ecosystems revealed by single-cell technologies. Trends Cancer.

[90] Jiang J (2021). Lactate modulates cellular metabolism through histone lactylation-mediated gene expression in non-small cell lung cancer. Front. Oncol..

[91] Yang K (2022). Lactate promotes macrophage HMGB1 lactylation, acetylation, and exosomal release in polymicrobial sepsis. Cell Death Differ..

[92] Xiong J (2022). Lactylation-driven METTL3-mediated RNA m(6)A modification promotes immunosuppression of tumor-infiltrating myeloid cells. Mol. Cell.

[93] Caielli S (2021). Erythroid mitochondrial retention triggers myeloid-dependent type I interferon in human SLE. Cell.

[94] Hagihara H (2021). Protein lactylation induced by neural excitation. Cell Rep..

[95] Xu, X. et al. Autophagic feedback-mediated degradation of IKKalpha requires CHK1- and p300/CBP-dependent acetylation of p53. *J. Cell Sci*. **133**, jcs246868 (2020). 10.1242/jcs.246868 33097607

[96] Manickavinayaham S (2019). E2F1 acetylation directs p300/CBP-mediated histone acetylation at DNA double-strand breaks to facilitate repair. Nat. Commun..

[97] Waddell, A. et al. Pharmacological inhibition of CBP/p300 blocks estrogen receptor alpha (ERalpha) function through suppressing enhancer H3K27 acetylation in luminal breast cancer. *Cancers* **13**, 2799 (2021). 10.3390/cancers13112799 PMC8200112 34199844

[98] Li L (2020). Glis1 facilitates induction of pluripotency via an epigenome-metabolome-epigenome signalling cascade. Nat. Metab..

[99] Moreno-Yruela C (2022). Class I histone deacetylases (HDAC1-3) are histone lysine delactylases. Sci. Adv..

[100] Mews P (2017). Acetyl-CoA synthetase regulates histone acetylation and hippocampal memory. Nature.

[101] Zhou W (2019). TIGAR promotes neural stem cell differentiation through acetyl-CoA-mediated histone acetylation. Cell Death Dis..

[102] Gaffney DO (2020). Non-enzymatic lysine lactoylation of glycolytic enzymes. Cell Chem. Biol..

[103] Rose IA, Nowick JS (2002). Methylglyoxal synthetase, enol-pyruvaldehyde, glutathione, and the glyoxalase system. J. Am. Chem. Soc..

[104] Rabbani N, Xue M, Thornalley PJ (2014). Activity, regulation, copy number and function in the glyoxalase system. Biochem. Soc. Trans..

[105] Pasti, A. P. et al. Human lactate dehydrogenase A undergoes allosteric transitions under pH conditions inducing the dissociation of the tetrameric enzyme. *Biosci. Rep*. **42**, BSR20212654 (2022). 10.1042/BSR20212654 PMC8799922 35048959

[106] Stolberg L (1982). d-Lactic acidosis due to abnormal gut flora: Diagnosis and treatment of two cases. N. Engl. J. Med..

[107] Ewaschuk JB, Naylor JM, Zello GA (2005). D-lactate in human and ruminant metabolism. J. Nutr..

[108] de Bari L (2002). D-Lactate transport and metabolism in rat liver mitochondria. Biochem. J..

[109] Flick MJ, Konieczny SF (2002). Identification of putative mammalian D-lactate dehydrogenase enzymes. Biochem. Biophys. Res. Commun..

[110] de Vrese M, Koppenhoefer B, Barth CA (1990). D-lactic acid metabolism after an oral load of DL-lactate. Clin. Nutr..

[111] Oh MS (1985). Metabolic utilization and renal handling of D-lactate in men. Metabolism.

[112] Schmitt M, Greten FR (2021). The inflammatory pathogenesis of colorectal cancer. Nat. Rev. Immunol..

[113] Rowley AH (2020). Understanding SARS-CoV-2-related multisystem inflammatory syndrome in children. Nat. Rev. Immunol..

[114] Frangogiannis NG (2014). The inflammatory response in myocardial injury, repair, and remodelling. Nat. Rev. Cardiol..

[115] Querol L, Devaux J, Rojas-Garcia R, Illa I (2017). Autoantibodies in chronic inflammatory neuropathies: Diagnostic and therapeutic implications. Nat. Rev. Neurol..

[116] Gao B, Ahmad MF, Nagy LE, Tsukamoto H (2019). Inflammatory pathways in alcoholic steatohepatitis. J. Hepatol..

[117] Afonina IS, Zhong Z, Karin M, Beyaert R (2017). Limiting inflammation-the negative regulation of NF-kappaB and the NLRP3 inflammasome. Nat. Immunol..

[118] Wullaert A, Bonnet MC, Pasparakis M (2011). NF-kappaB in the regulation of epithelial homeostasis and inflammation. Cell Res..

[119] Hinz M, Arslan SC, Scheidereit C (2012). It takes two to tango: IkappaBs, the multifunctional partners of NF-kappaB. Immunol. Rev..

[120] Miao F, Shan C, Ning D (2021). Walnut oil alleviates LPS-induced intestinal epithelial cells injury by inhibiting TLR4/MyD88/NF-kappaB pathway activation. J. Food Biochem..

[121] Qi, S. et al. Silencing of PTX3 alleviates LPS-induced inflammatory pain by regulating TLR4/NF-kappaB signaling pathway in mice. *Biosci. Rep*. **40**, BSR20194208 (2020). 10.1042/BSR20194208 PMC7000368 31957804

[122] Abebayehu D (2019). Lactic acid suppresses IgE-mediated mast cell function in vitro and in vivo. Cell Immunol..

[123] Peter K (2015). Lactic acid delays the inflammatory response of human monocytes. Biochem. Biophys. Res. Commun..

[124] Yang K (2020). Lactate suppresses macrophage pro-inflammatory response to LPS stimulation by inhibition of YAP and NF-kappaB activation via GPR81-mediated signaling. Front. Immunol..

[125] Arango Duque G, Descoteaux A (2014). Macrophage cytokines: Involvement in immunity and infectious diseases. Front. Immunol..

[126] Ubil E (2018). Tumor-secreted Pros1 inhibits macrophage M1 polarization to reduce antitumor immune response. J. Clin. Invest..

[127] Sharif O, Brunner JS, Vogel A, Schabbauer G (2019). Macrophage rewiring by nutrient associated PI3K dependent pathways. Front. Immunol..

[128] Galvan-Pena S, O’Neill LA (2014). Metabolic reprograming in macrophage polarization. Front. Immunol..

[129] Gharib SA (2019). Transcriptional and functional diversity of human macrophage repolarization. J. Allergy Clin. Immunol..

[130] Kumar V (2018). Targeting macrophage immunometabolism: Dawn in the darkness of sepsis. Int. Immunopharmacol..

[131] Costa Leite T (2007). Lactate favours the dissociation of skeletal muscle 6-phosphofructo-1-kinase tetramers down-regulating the enzyme and muscle glycolysis. Biochem. J..

[132] Zhang J (2020). Endothelial lactate controls muscle regeneration from ischemia by inducing M2-like macrophage polarization. Cell Metab..

[133] Selleri S (2016). Human mesenchymal stromal cell-secreted lactate induces M2-macrophage differentiation by metabolic reprogramming. Oncotarget.

[134] Kolkhir P (2022). Understanding human mast cells: Lesson from therapies for allergic and non-allergic diseases. Nat. Rev. Immunol..

[135] Syed M (2021). Lactic acid suppresses MRGPRX2 mediated mast cell responses. Cell Immunol..

[136] Ranganathan P (2018). GPR81, a cell-surface receptor for lactate, regulates intestinal homeostasis and protects mice from experimental colitis. J. Immunol..

[137] Xu J (2021). Lactate up-regulates the expression of PD-L1 in kidney and causes immunosuppression in septic Acute Renal Injury. J. Microbiol. Immunol. Infect..

[138] Shan T (2020). M2TAM subsets altered by lactic acid promote Tcell apoptosis through the PDL1/PD1 pathway. Oncol. Rep..

[139] Hoque R (2014). Lactate reduces liver and pancreatic injury in Toll-like receptor- and inflammasome-mediated inflammation via GPR81-mediated suppression of innate immunity. Gastroenterology.

[140] Caslin HL (2019). Lactic acid inhibits lipopolysaccharide-induced mast cell function by limiting glycolysis and ATP availability. J. Immunol..

[141] Rajendran P (2018). The multifaceted link between inflammation and human diseases. J. Cell Physiol..

[142] Droge W, Roth S, Altmann A, Mihm S (1987). Regulation of T-cell functions by L-lactate. Cell Immunol..

[143] Haas R (2015). Lactate regulates metabolic and pro-inflammatory circuits in control of T cell migration and effector functions. PLoS Biol..

[144] Souto-Carneiro MM (2020). Effect of increased lactate dehydrogenase A activity and aerobic glycolysis on the proinflammatory profile of autoimmune CD8+T cells in rheumatoid arthritis. Arthritis Rheumatol..

[145] Xie N (2017). Metabolic characterization and RNA profiling reveal glycolytic dependence of profibrotic phenotype of alveolar macrophages in lung fibrosis. Am. J. Physiol. Lung Cell Mol. Physiol..

[146] Reyfman PA (2019). Single-cell transcriptomic analysis of human lung provides insights into the pathobiology of pulmonary fibrosis. Am. J. Respir. Crit. Care Med..

[147] Imtiyaz HZ (2010). Hypoxia-inducible factor 2alpha regulates macrophage function in mouse models of acute and tumor inflammation. J. Clin. Invest..

[148] Carmona-Fontaine C (2017). Metabolic origins of spatial organization in the tumor microenvironment. Proc. Natl Acad. Sci. USA.

[149] De Vlaeminck Y (2019). Single-domain antibody fusion proteins can target and shuttle functional proteins into macrophage mannose receptor expressing macrophages. J. Control Release.

[150] Guo L (2019). Induction of breast cancer stem cells by M1 macrophages through Lin-28B-let-7-HMGA2 axis. Cancer Lett..

[151] Jackute J (2018). Distribution of M1 and M2 macrophages in tumor islets and stroma in relation to prognosis of non-small cell lung cancer. BMC Immunol..

[152] Chen Y, Zhang S, Wang Q, Zhang X (2017). Tumor-recruited M2 macrophages promote gastric and breast cancer metastasis via M2 macrophage-secreted CHI3L1 protein. J. Hematol. Oncol..

[153] Li W (2019). Gastric cancer-derived mesenchymal stromal cells trigger M2 macrophage polarization that promotes metastasis and EMT in gastric cancer. Cell Death Dis..

[154] Zhao S (2020). Tumor-derived exosomal miR-934 induces macrophage M2 polarization to promote liver metastasis of colorectal cancer. J. Hematol. Oncol..

[155] Mu X (2018). Tumor-derived lactate induces M2 macrophage polarization via the activation of the ERK/STAT3 signaling pathway in breast cancer. Cell Cycle.

[156] Colegio OR (2014). Functional polarization of tumour-associated macrophages by tumour-derived lactic acid. Nature.

[157] Peters A (2011). Th17 cells induce ectopic lymphoid follicles in central nervous system tissue inflammation. Immunity.

[158] Angelin A (2017). Foxp3 reprograms T cell metabolism to function in low-glucose, high-lactate environments. Cell Metab..

[159] Ivashkiv LB (2020). The hypoxia-lactate axis tempers inflammation. Nat. Rev. Immunol..

[160] Errea A (2016). Lactate inhibits the pro-inflammatory response and metabolic reprogramming in murine macrophages in a GPR81-independent manner. PLoS One.

[161] Samuvel DJ (2009). Lactate boosts TLR4 signaling and NF-kappaB pathway-mediated gene transcription in macrophages via monocarboxylate transporters and MD-2 up-regulation. J. Immunol..

[162] Fischbeck AJ (2020). Tumor lactic acidosis: Protecting tumor by inhibiting cytotoxic activity through motility arrest and bioenergetic silencing. Front. Oncol..

[163] Watson MJ (2021). Metabolic support of tumour-infiltrating regulatory T cells by lactic acid. Nature.

[164] Paolini L (2020). Lactic acidosis together with GM-CSF and M-CSF induces human macrophages toward an inflammatory protumor phenotype. Cancer Immunol. Res..

[165] Fernandez SF (2013). Low pH environmental stress inhibits LPS and LTA-stimulated proinflammatory cytokine production in rat alveolar macrophages. Biomed. Res. Int..

[166] Gottfried E (2006). Tumor-derived lactic acid modulates dendritic cell activation and antigen expression. Blood.

[167] Noormandi A, Khalili H, Mohammadi M, Abdollahi A (2020). Effect of magnesium supplementation on lactate clearance in critically ill patients with severe sepsis: A randomized clinical trial. Eur. J. Clin. Pharm..

[168] Wani SJ (2020). Combination of vitamin C, thiamine and hydrocortisone added to standard treatment in the management of sepsis: Results from an open label randomised controlled clinical trial and a review of the literature. Infect. Dis..

[169] Zhang Y (2019). Efficacy of continuous renal replacement on acute renal injury developed in severe sepsis. J. Biol. Regul. Homeost. Agents.

[170] Jones AE (2010). Lactate clearance vs central venous oxygen saturation as goals of early sepsis therapy: A randomized clinical trial. JAMA.

[171] Lee SM (2015). Lactate clearance and vasopressor seem to be predictors for mortality in severe sepsis patients with lactic acidosis supplementing sodium bicarbonate: A retrospective analysis. PLoS One.

[172] Zhou X (2017). Use of stepwise lactate kinetics-oriented hemodynamic therapy could improve the clinical outcomes of patients with sepsis-associated hyperlactatemia. Crit. Care.

[173] Chen H (2022). Early lactate-guided resuscitation of elderly septic patients. J. Intensive Care Med..

[174] During J (2018). Lactate, lactate clearance and outcome after cardiac arrest: A post-hoc analysis of the TTM-Trial. Acta Anaesthesiol. Scand..

[175] Besnier E (2020). Hypertonic sodium lactate improves microcirculation, cardiac function, and inflammation in a rat model of sepsis. Crit. Care.

[176] van Hall G (2010). Lactate kinetics in human tissues at rest and during exercise. Acta Physiol..

[177] Dienel GA (2012). Brain lactate metabolism: The discoveries and the controversies. J. Cereb. Blood Flow. Metab..

[178] Halestrap AP (2012). The monocarboxylate transporter family-Structure and functional characterization. IUBMB Life.

[179] Descalzi G (2019). Lactate from astrocytes fuels learning-induced mRNA translation in excitatory and inhibitory neurons. Commun. Biol..

[180] Veloz Castillo, M. F., Magistretti, P. J. & Cali, C. l-Lactate: Food for thoughts, memory, and behavior. *Metabolites* **11**, 548 (2021). 10.3390/metabo11080548 PMC8398236 34436491

[181] Yang J (2014). Lactate promotes plasticity gene expression by potentiating NMDA signaling in neurons. Proc. Natl Acad. Sci. USA.

[182] Wang J (2019). Brain endothelial cells maintain lactate homeostasis and control adult hippocampal neurogenesis. Cell Stem Cell.

[183] Alberini CM (2018). Astrocyte glycogen and lactate: New insights into learning and memory mechanisms. Glia.

[184] Lu WT (2019). Curcumin ameliorates memory deficits by enhancing lactate content and MCT2 expression in APP/PS1 transgenic mouse model of Alzheimer’s disease. Anat. Rec..

[185] El Hayek L (2019). Lactate mediates the effects of exercise on learning and memory through SIRT1-dependent activation of hippocampal brain-derived neurotrophic factor (BDNF). J. Neurosci..

[186] Morland C (2015). The lactate receptor, G-protein-coupled receptor 81/hydroxycarboxylic acid receptor 1: Expression and action in brain. J. Neurosci. Res..

[187] Wilkinson HN, Hardman MJ (2020). Wound healing: Cellular mechanisms and pathological outcomes. Open Biol..

[188] Haller, H. L. et al. Oxygen, pH, lactate, and metabolism—How old knowledge and new insights might be combined for new wound treatment. *Medicina* **57**, 1190 (2021). 10.3390/medicina57111190 PMC8617754 34833408

[189] DeBerardinis RJ, Chandel NS (2020). We need to talk about the Warburg effect. Nat. Metab..

[190] Trabold O (2003). Lactate and oxygen constitute a fundamental regulatory mechanism in wound healing. Wound Repair Regen..

[191] Hunt TK, Aslam R, Hussain Z, Beckert S (2008). Lactate, with oxygen, incites angiogenesis. Adv. Exp. Med. Biol..

[192] Hunt TK (2007). Aerobically derived lactate stimulates revascularization and tissue repair via redox mechanisms. Antioxid. Redox Signal.

[193] Hunt TK, Conolly WB, Aronson SB, Goldstein P (1978). Anaerobic metabolism and wound healing: an hypothesis for the initiation and cessation of collagen synthesis in wounds. Am. J. Surg..

[194] Milovanova TN (2008). Lactate stimulates vasculogenic stem cells via the thioredoxin system and engages an autocrine activation loop involving hypoxia-inducible factor 1. Mol. Cell Biol..

[195] Liu Q (2004). A Fenton reaction at the endoplasmic reticulum is involved in the redox control of hypoxia-inducible gene expression. Proc. Natl Acad. Sci. USA.

[196] Vural E (2010). Skin graft take rates, granulation, and epithelialization: Dependence on myeloid cell hypoxia-inducible factor 1alpha. Arch. Otolaryngol. Head. Neck Surg..

[197] Savitri C (2022). M2 macrophage-derived concentrated conditioned media significantly improves skin wound healing. Tissue Eng. Regen. Med..

[198] Zhang SM (2021). M2-polarized macrophages mediate wound healing by regulating connective tissue growth factor via AKT, ERK1/2, and STAT3 signaling pathways. Mol. Biol. Rep..

[199] Gu S (2020). AKT3 deficiency in M2 macrophages impairs cutaneous wound healing by disrupting tissue remodeling. Aging.

[200] Fu J (2020). Quercetin promotes diabetic wound healing via switching macrophages from M1 to M2 polarization. J. Surg. Res..

[201] Vagesjo E (2018). Accelerated wound healing in mice by on-site production and delivery of CXCL12 by transformed lactic acid bacteria. Proc. Natl Acad. Sci. USA.

[202] Ohnstedt, E. et al. Accelerated wound healing in minipigs by on-site production and delivery of CXCL12 by transformed lactic acid bacteria. *Pharmaceutics* **14**, 229 (2022). 10.3390/pharmaceutics14020229 PMC8876577 35213962

[203] Davis FM, Gallagher K (2018). Time heals all wounds… but wounds heal faster with lactobacillus. Cell Host Microbe.

[204] Kinney HC (2005). Hypoxic-ischemic brain injury in infants with congenital heart disease dying after cardiac surgery. Acta Neuropathol..

[205] Baartscheer A (2003). Increased Na+/H+-exchange activity is the cause of increased [Na+]i and underlies disturbed calcium handling in the rabbit pressure and volume overload heart failure model. Cardiovasc. Res..

[206] Slepkov ER, Rainey JK, Sykes BD, Fliegel L (2007). Structural and functional analysis of the Na+/H+exchanger. Biochem. J..

[207] Andersson AK, Ronnback L, Hansson E (2005). Lactate induces tumour necrosis factor-alpha, interleukin-6 and interleukin-1beta release in microglial and astroglial-enriched primary cultures. J. Neurochem..

[208] Shen Z (2015). Inhibition of G protein-coupled receptor 81 (GPR81) protects against ischemic brain injury. CNS Neurosci. Ther..

[209] Berthet C, Castillo X, Magistretti PJ, Hirt L (2012). New evidence of neuroprotection by lactate after transient focal cerebral ischaemia: Extended benefit after intracerebroventricular injection and efficacy of intravenous administration. Cerebrovasc. Dis..

[210] Tassinari ID (2020). Lactate administration reduces brain injury and ameliorates behavioral outcomes following neonatal hypoxia-ischemia. Neuroscience.

[211] Haege, E. R., Huang, H. C. & Huang, C. C. Identification of lactate as a cardiac protectant by inhibiting inflammation and cardiac hypertrophy using a zebrafish acute heart failure model. *Pharmaceuticals* **14**, 261 (2021). 10.3390/ph14030261 PMC7999541 33803943

[212] Molstrom S (2021). Bedside microdialysis for detection of early brain injury after out-of-hospital cardiac arrest. Sci. Rep..

[213] Theodoraki K (2006). Transhepatic lactate gradient in relation to liver ischemia/reperfusion injury during major hepatectomies. Liver Transpl..

[214] Marion DW (2002). Effect of hyperventilation on extracellular concentrations of glutamate, lactate, pyruvate, and local cerebral blood flow in patients with severe traumatic brain injury. Crit. Care Med..

[215] Frydland M (2019). Lactate is a prognostic factor in patients admitted with suspected ST-elevation myocardial infarction. Shock.

[216] Hajjar LA (2013). High lactate levels are predictors of major complications after cardiac surgery. J. Thorac. Cardiovasc. Surg..

[217] Li B, Chen R, Huang R, Luo W (2009). Clinical benefit of cardiac ischemic postconditioning in corrections of tetralogy of Fallot. Interact. Cardiovasc. Thorac. Surg..

[218] Li L (2018). TAp73-induced phosphofructokinase-1 transcription promotes the Warburg effect and enhances cell proliferation. Nat. Commun..

[219] Lunt SY, Vander Heiden MG (2011). Aerobic glycolysis: Meeting the metabolic requirements of cell proliferation. Annu. Rev. Cell Dev. Biol..

[220] Roland CL (2014). Cell surface lactate receptor GPR81 is crucial for cancer cell survival. Cancer Res..

[221] Brown TP (2020). The lactate receptor GPR81 promotes breast cancer growth via a paracrine mechanism involving antigen-presenting cells in the tumor microenvironment. Oncogene.

[222] Feng J (2017). Tumor cell-derived lactate induces TAZ-dependent upregulation of PD-L1 through GPR81 in human lung cancer cells. Oncogene.

[223] Estrella V (2013). Acidity generated by the tumor microenvironment drives local invasion. Cancer Res..

[224] Parks SK, Chiche J, Pouyssegur J (2013). Disrupting proton dynamics and energy metabolism for cancer therapy. Nat. Rev. Cancer.

[225] Martinez-Zaguilan R (1999). pH and drug resistance. I. Functional expression of plasmalemmal V-type H+-ATPase in drug-resistant human breast carcinoma cell lines. Biochem. Pharm..

[226] Wang H (2020). LAMC2 modulates the acidity of microenvironments to promote invasion and migration of pancreatic cancer cells via regulating AKT-dependent NHE1 activity. Exp. Cell Res..

[227] Mo L (2021). Shikonin suppresses the epithelial-to-mesenchymal transition by downregulating NHE1 in bladder cancer cells. J. Cancer.

[228] Sun Z (2020). NHE1 mediates 5-Fu resistance in gastric cancer via STAT3 signaling pathway. Onco Targets Ther..

[229] Xie R (2017). NHE1 is upregulated in gastric cancer and regulates gastric cancer cell proliferation, migration, and invasion. Oncol. Rep..

[230] Lee S (2018). Na(+),HCO3(-)-cotransporter NBCn1 (Slc4a7) accelerates ErbB2-induced breast cancer development and tumor growth in mice. Oncogene.

[231] Lee S (2016). Disrupting Na(+), HCO(3)(-)-cotransporter NBCn1 (Slc4a7) delays murine breast cancer development. Oncogene.

[232] Lauritzen G (2012). The Na+/H+exchanger NHE1, but not the Na+, HCO3(-) cotransporter NBCn1, regulates motility of MCF7 breast cancer cells expressing constitutively active ErbB2. Cancer Lett..

[233] De Milito A, Fais S (2005). Tumor acidity, chemoresistance, and proton pump inhibitors. Future Oncol..

[234] Flinck M (2018). The acid-base transport proteins NHE1 and NBCn1 regulate cell cycle progression in human breast cancer cells. Cell Cycle.

[235] Andersen AP (2018). The net acid extruders NHE1, NBCn1, and MCT4 promote mammary tumor growth through distinct but overlapping mechanisms. Int. J. Cancer.

[236] Vegran F (2011). Lactate influx through the endothelial cell monocarboxylate transporter MCT1 supports an NF-kappaB/IL-8 pathway that drives tumor angiogenesis. Cancer Res..

[237] Lee DC (2015). A lactate-induced response to hypoxia. Cell.

[238] Li L (2020). Hypoxia-induced GBE1 expression promotes tumor progression through metabolic reprogramming in lung adenocarcinoma. Signal Transduct. Target Ther..

[239] Park MJ (2018). HIF1-alpha regulates acinar cell function and response to injury in mouse pancreas. Gastroenterology.

[240] Lu H (2005). Reversible inactivation of HIF-1 prolyl hydroxylases allows cell metabolism to control basal HIF-1. J. Biol. Chem..

[241] Vollmann-Zwerenz, A. et al. Tumor cell invasion in glioblastoma. *Int. J. Mol. Sci*. **21**, 1932 (2020). 10.3390/ijms21061932 PMC7139341 32178267

[242] Savagner P (2015). Epithelial-mesenchymal transitions: From cell plasticity to concept elasticity. Curr. Top. Dev. Biol..

[243] Sahai E (2020). A framework for advancing our understanding of cancer-associated fibroblasts. Nat. Rev. Cancer.

[244] Schulz MC, Wagenbrett L, Schwerdt G, Gekle M (2018). Influence of extracellular acidosis on matrix protein homeostasis in tumour cells and fibroblasts. Adv. Exp. Med. Biol..

[245] Ogunwobi OO, Wang T, Zhang L, Liu C (2012). Cyclooxygenase-2 and Akt mediate multiple growth-factor-induced epithelial-mesenchymal transition in human hepatocellular carcinoma. J. Gastroenterol. Hepatol..

[246] Ahn JH, Choi YS, Choi JH (2015). Leptin promotes human endometriotic cell migration and invasion by up-regulating MMP-2 through the JAK2/STAT3 signaling pathway. Mol. Hum. Reprod..

[247] Ohno Y (2018). Lactate increases myotube diameter via activation of MEK/ERK pathway in C2C12 cells. Acta Physiol..

[248] Lagares-Tena L (2016). Caveolin-1 promotes Ewing sarcoma metastasis regulating MMP-9 expression through MAPK/ERK pathway. Oncotarget.

[249] Niu D (2021). Lactic acid in tumor invasion. Clin. Chim. Acta.

[250] Boedtkjer E, Pedersen SF (2020). The acidic tumor microenvironment as a driver of cancer. Annu. Rev. Physiol..

[251] Nakagawa Y (2015). Effects of extracellular pH and hypoxia on the function and development of antigen-specific cytotoxic T lymphocytes. Immunol. Lett..

[252] Fischer K (2007). Inhibitory effect of tumor cell-derived lactic acid on human T cells. Blood.

[253] Almeida L (2021). CD4(+) T-cell differentiation and function: Unifying glycolysis, fatty acid oxidation, polyamines NAD mitochondria. J. Allergy Clin. Immunol..

[254] Peng M (2016). Aerobic glycolysis promotes T helper 1 cell differentiation through an epigenetic mechanism. Science.

[255] Frauwirth KA (2002). The CD28 signaling pathway regulates glucose metabolism. Immunity.

[256] Teijeira A (2019). Metabolic consequences of T-cell costimulation in anticancer immunity. Cancer Immunol. Res..

[257] Xu K (2021). Glycolysis fuels phosphoinositide 3-kinase signaling to bolster T cell immunity. Science.

[258] Mendler AN (2012). Tumor lactic acidosis suppresses CTL function by inhibition of p38 and JNK/c-Jun activation. Int. J. Cancer.

[259] Calcinotto A (2012). Modulation of microenvironment acidity reverses anergy in human and murine tumor-infiltrating T lymphocytes. Cancer Res..

[260] Navarro F (2022). Overcoming T cell dysfunction in acidic pH to enhance adoptive T cell transfer immunotherapy. Oncoimmunology.

[261] Noman MZ (2014). PD-L1 is a novel direct target of HIF-1alpha, and its blockade under hypoxia enhanced MDSC-mediated T cell activation. J. Exp. Med..

[262] Bian Y (2020). Cancer SLC43A2 alters T cell methionine metabolism and histone methylation. Nature.

[263] Brand A (2016). LDHA-associated lactic acid production blunts tumor immunosurveillance by T and NK cells. Cell Metab..

[264] Chi H (2012). Regulation and function of mTOR signalling in T cell fate decisions. Nat. Rev. Immunol..

[265] Erra Diaz F, Dantas E, Geffner J (2018). Unravelling the interplay between extracellular acidosis and immune cells. Mediators Inflamm..

[266] Gu J (2022). Tumor metabolite lactate promotes tumorigenesis by modulating MOESIN lactylation and enhancing TGF-beta signaling in regulatory T cells. Cell Rep..

[267] Gabrilovich DI, Ostrand-Rosenberg S, Bronte V (2012). Coordinated regulation of myeloid cells by tumours. Nat. Rev. Immunol..

[268] Wang R (2011). Increased IL-10 mRNA expression in tumor-associated macrophage correlated with late stage of lung cancer. J. Exp. Clin. Cancer Res..

[269] Pollari M (2018). PD-L1(+) tumor-associated macrophages and PD-1(+) tumor-infiltrating lymphocytes predict survival in primary testicular lymphoma. Haematologica.

[270] Rodriguez PC (2004). Arginase I production in the tumor microenvironment by mature myeloid cells inhibits T-cell receptor expression and antigen-specific T-cell responses. Cancer Res..

[271] Yang L (2014). PKM2 regulates the Warburg effect and promotes HMGB1 release in sepsis. Nat. Commun..

[272] Wang S, Zhang Y (2020). HMGB1 in inflammation and cancer. J. Hematol. Oncol..

[273] Zhang QY (2015). Autophagy-mediated HMGB1 release promotes gastric cancer cell survival via RAGE activation of extracellular signal-regulated kinases 1/2. Oncol. Rep..

[274] Pistoia V, Pezzolo A (2016). Involvement of HMGB1 in resistance to tumor vessel-targeted, monoclonal antibody-based immunotherapy. J. Immunol. Res..

[275] Guan X, Rodriguez-Cruz V, Morris ME (2019). Cellular uptake of MCT1 inhibitors AR-C155858 and AZD3965 and their effects on MCT-mediated transport of L-lactate in murine 4T1 breast tumor cancer cells. AAPS J..

[276] Vlachostergios PJ, Oikonomou KG, Gibilaro E, Apergis G (2015). Elevated lactic acid is a negative prognostic factor in metastatic lung cancer. Cancer Biomark..

[277] Wei Y (2018). Prognostic significance of serum lactic acid, lactate dehydrogenase, and albumin levels in patients with metastatic colorectal cancer. Biomed. Res. Int..

[278] Walenta S (2000). High lactate levels predict likelihood of metastases, tumor recurrence, and restricted patient survival in human cervical cancers. Cancer Res..

[279] Matsumura A (2005). Non-invasive quantification of lactate by proton MR spectroscopy and its clinical applications. Clin. Neurol. Neurosurg..

[280] Charles-Edwards GD (2010). Non-invasive detection and quantification of human foetal brain lactate in utero by magnetic resonance spectroscopy. Prenat. Diagn..

[281] Isobe T (2007). Lactate quantification by proton magnetic resonance spectroscopy using a clinical MRI machine: a basic study. Australas. Radio..

[282] Wang ZJ (2019). Hyperpolarized (13)C MRI: State of the art and future directions. Radiology.

[283] Cheung SM (2020). Lactate concentration in breast cancer using advanced magnetic resonance spectroscopy. Br. J. Cancer.

[284] Giskeodegard GF (2012). Lactate and glycine-potential MR biomarkers of prognosis in estrogen receptor-positive breast cancers. NMR Biomed..

[285] Xu HN (2014). Is higher lactate an indicator of tumor metastatic risk? A pilot MRS study using hyperpolarized (13)C-pyruvate. Acad. Radio..

[286] Wolpaw AJ, Dang CV (2018). Exploiting metabolic vulnerabilities of cancer with precision and accuracy. Trends Cell Biol..

[287] Miao P (2013). Lactate dehydrogenase A in cancer: A promising target for diagnosis and therapy. IUBMB Life.

[288] Ippolito L, Morandi A, Giannoni E, Chiarugi P (2019). Lactate: A metabolic driver in the tumour landscape. Trends Biochem. Sci..

[289] Maekawa M (2003). Promoter hypermethylation in cancer silences LDHB, eliminating lactate dehydrogenase isoenzymes 1–4. Clin. Chem..

[290] Leiblich A (2006). Lactate dehydrogenase-B is silenced by promoter hypermethylation in human prostate cancer. Oncogene.

[291] Brown NJ (2013). Lactate dehydrogenase-B is silenced by promoter methylation in a high frequency of human breast cancers. PLoS One.

[292] Cui J (2015). Suppressed expression of LDHB promotes pancreatic cancer progression via inducing glycolytic phenotype. Med. Oncol..

[293] Wu H (2021). Lactate dehydrogenases amplify reactive oxygen species in cancer cells in response to oxidative stimuli. Signal Transduct. Target Ther..

[294] Maeda, M. et al. Genetic and drug inhibition of LDH-A: Effects on murine gliomas. *Cancers* **14**, 2306 (2022). 10.3390/cancers14092306 PMC9105502 35565435

[295] Zhang, W. et al. Inhibition of LDHA suppresses cell proliferation and increases mitochondrial apoptosis via the JNK signaling pathway in cervical cancer cells. *Oncol Rep*. **47**, 77 (2022). 10.3892/or.2022.8288 PMC8892607 35191522

[296] Jin L (2017). Phosphorylation-mediated activation of LDHA promotes cancer cell invasion and tumour metastasis. Oncogene.

[297] Hou X (2021). LDHA induces EMT gene transcription and regulates autophagy to promote the metastasis and tumorigenesis of papillary thyroid carcinoma. Cell Death Dis..

[298] Le A (2010). Inhibition of lactate dehydrogenase A induces oxidative stress and inhibits tumor progression. Proc. Natl Acad. Sci. USA.

[299] Mohammad, G. H. et al. Targeting pyruvate kinase M2 and lactate dehydrogenase A is an effective combination strategy for the treatment of pancreatic cancer. *Cancers* **11**, 1372 (2019). 10.3390/cancers11091372 PMC6770573 31527446

[300] Manerba M (2012). Galloflavin (CAS 568-80-9): A novel inhibitor of lactate dehydrogenase. ChemMedChem.

[301] Vettraino M, Manerba M, Govoni M, Di Stefano G (2013). Galloflavin suppresses lactate dehydrogenase activity and causes MYC downregulation in Burkitt lymphoma cells through NAD/NADH-dependent inhibition of sirtuin-1. Anticancer Drugs.

[302] Cui B (2019). Stress-induced epinephrine enhances lactate dehydrogenase A and promotes breast cancer stem-like cells. J. Clin. Invest..

[303] Maekawa M, Sudo K, Kanno T, Li SS (1990). Molecular characterization of genetic mutation in human lactate dehydrogenase-A (M) deficiency. Biochem. Biophys. Res. Commun..

[304] Kanno T (1988). Lactate dehydrogenase M-subunit deficiency: A new type of hereditary exertional myopathy. Clin. Chim. Acta.

[305] Elia I (2019). Breast cancer cells rely on environmental pyruvate to shape the metastatic niche. Nature.

[306] Doherty, J. et al. Blocking lactate export by inhibiting the Myc target MCT1 Disables glycolysis and glutathione synthesis. *Cancer Res.* **74**, 908–920 (2014). 10.1158/0008-5472.CAN-13-2034 PMC3946415 24285728

[307] Gan L (2016). Metabolic targeting of oncogene MYC by selective activation of the proton-coupled monocarboxylate family of transporters. Oncogene.

[308] Khan A (2020). Targeting metabolic activity in high-risk neuroblastoma through Monocarboxylate Transporter 1 (MCT1) inhibition. Oncogene.

[309] Huang HK (2020). Isoorientin decreases cell migration via decreasing functional activity and molecular expression of proton-linked monocarboxylate transporters in human lung cancer cells. Am. J. Chin. Med..

[310] Wang N (2021). Structural basis of human monocarboxylate transporter 1 inhibition by anti-cancer drug candidates. Cell.

[311] Buyse, C. et al. Evaluation of syrosingopine, an MCT inhibitor, as potential modulator of tumor metabolism and extracellular acidification. *Metabolites*. **12**, 557 (2022). 10.3390/metabo12060557 PMC9230831 35736489

[312] Benjamin D (2016). Syrosingopine sensitizes cancer cells to killing by metformin. Sci. Adv..

[313] Benyahia, Z. et al. In vitro and in vivo characterization of MCT1 inhibitor AZD3965 confirms preclinical safety compatible with breast cancer treatment. *Cancers* **13**, 569 (2021). 10.3390/cancers13030569 PMC7867268 33540599

[314] Dana P (2020). CD147 augmented monocarboxylate transporter-1/4 expression through modulation of the Akt-FoxO3-NF-kappaB pathway promotes cholangiocarcinoma migration and invasion. Cell Oncol..

[315] Zhang D (2014). 2-Deoxy-D-glucose targeting of glucose metabolism in cancer cells as a potential therapy. Cancer Lett..

[316] Raez LE (2013). A phase I dose-escalation trial of 2-deoxy-D-glucose alone or combined with docetaxel in patients with advanced solid tumors. Cancer Chemother. Pharm..

[317] Bizjak M (2017). Combined treatment with Metformin and 2-deoxy glucose induces detachment of viable MDA-MB-231 breast cancer cells in vitro. Sci. Rep..

[318] Sukumar M (2013). Inhibiting glycolytic metabolism enhances CD8+T cell memory and antitumor function. J. Clin. Invest..

[319] Dunbar EM (2014). Phase 1 trial of dichloroacetate (DCA) in adults with recurrent malignant brain tumors. Invest. N. Drugs.

[320] Powell SF (2022). Phase II study of dichloroacetate, an inhibitor of pyruvate dehydrogenase, in combination with chemoradiotherapy for unresected, locally advanced head and neck squamous cell carcinoma. Invest. N. Drugs.

[321] Li M (2022). miR-564: A potential regulator of vascular smooth muscle cells and therapeutic target for aortic dissection. J. Mol. Cell Cardiol..

[322] Yang Y (2022). The lncRNA punisher regulates apoptosis and mitochondrial homeostasis of vascular smooth muscle cells via targeting miR-664a-5p and OPA1. Oxid. Med. Cell Longev..

[323] Li X (2022). Multistage-responsive nanocomplexes attenuate ulcerative colitis by improving the accumulation and distribution of oral nucleic acid drugs in the colon. ACS Appl. Mater. Interfaces.

[324] Qi HY (2022). Glucose-responsive nanogels efficiently maintain the stability and activity of therapeutic enzymes. Nanotechnol. Rev..

[325] Dancy BM, Cole PA (2015). Protein lysine acetylation by p300/CBP. Chem. Rev..

[326] Huang, H. et al. The regulatory enzymes and protein substrates for the lysine beta-hydroxybutyrylation pathway. *Sci Adv*. **7**, eabe2771 (2021). 10.1126/sciadv.abe2771 PMC7904266 33627428

[327] Sabari BR, Zhang D, Allis CD, Zhao Y (2017). Metabolic regulation of gene expression through histone acylations. Nat. Rev. Mol. Cell Biol..

[328] Yang Y (2021). Global insights into lysine acylomes reveal crosstalk between lysine acetylation and succinylation in streptomyces coelicolor metabolic pathways. Mol. Cell Proteom..

[329] Meng X, Baine JM, Yan T, Wang S (2021). Comprehensive analysis of lysine lactylation in rice (Oryza sativa) grains. J. Agric Food Chem..

[330] Shvedunova M, Akhtar A (2022). Modulation of cellular processes by histone and non-histone protein acetylation. Nat. Rev. Mol. Cell Biol..

[331] James AM (2017). Non-enzymatic N-acetylation of lysine residues by acetylCoA often occurs via a proximal S-acetylated thiol intermediate sensitive to glyoxalase II. Cell Rep..

[332] Gao M, Zhang N, Liang W (2020). Systematic analysis of lysine lactylation in the plant fungal pathogen Botrytis cinerea. Front. Microbiol..

