# Correction: Lactate metabolism in human health and disease

**DOI:** 10.1038/s41392-022-01206-5

**Published:** 2022-10-31

**Authors:** Xiaolu Li, Yanyan Yang, Bei Zhang, Xiaotong Lin, Xiuxiu Fu, Yi An, Yulin Zou, Jian-Xun Wang, Zhibin Wang, Tao Yu

**Affiliations:** 1 Center for Regenerative Medicine, Institute for Translational Medicine, The Affiliated Hospital of Qingdao University; Department of Cardiac Ultrasound, The Affiliated Hospital of Qingdao University, No. 16 Jiangsu Road, Qingdao, 266000 China; 2 Department of Immunology, School of Basic Medicine, Qingdao University, Qingdao, 266071 China; 3 Department of Respiratory Medicine, Qingdao Municipal Hospital, Qingdao, 266011 China; 4 Department of Cardiac Ultrasound, The Affiliated Hospital of Qingdao University, No. 16 Jiangsu Road, Qingdao, 266000 China; 5 Department of Cardiology, The Affiliated Hospital of Qingdao University, No. 1677 Wutaishan Road, Qingdao, 266555 China

**Keywords:** Epigenetics, Cancer

Correction to: *Signal Transduction and Targeted Therapy* 10.1038/s41392-022-01151-3, published online 01 September 2022

After online publication of the article^1^, the authors noticed one inadvertent mistake occurred in introduction that needs to be corrected. The correct text was provided as follows. The original article has been corrected.

In the 1920s, Otto Warburg observed for the first time that tumors consume more glucose than surrounding normal tissue, leading him to propose the phenomenon of aerobic glycolysis, wherein glucose can be fermented to produce lactate instead of carbon dioxide, even in the presence of oxygen; this phenomenon is now known as the Warburg effect.
